# Comparison Between Decitabine and Azacitidine for Patients With Acute Myeloid Leukemia and Higher-Risk Myelodysplastic Syndrome: A Systematic Review and Network Meta-Analysis

**DOI:** 10.3389/fphar.2021.701690

**Published:** 2021-08-17

**Authors:** Jiale Ma, Zheng Ge

**Affiliations:** ^1^ Department of Hematology, Zhongda Hospital, School of Medicine, Southeast University, Institute of Hematology Southeast University, Nanjing, China; ^2^ Department of Hematology, Xuzhou Central Hospital, Xuzhou, China

**Keywords:** decitabine, azacitidine, acute myeloid leukemia, higher-risk myelodysplastic syndrome, network meta-analysis

## Abstract

**Background:** The hypomethylating agents (HMAs) azacitidine (AZA) and decitabine (DAC) have been widely used in patients with acute myeloid leukemia (AML) and higher-risk myelodysplastic syndrome (HR-MDS). However, few direct clinical trials have been carried out to compare the efficacy and adverse events (AEs) between these two agents. The clinical choice between them is controversial. A systematic review and network meta-analysis (NMA) was performed to compare the efficacy, safety, and survival of DAC and AZA in AML and HR-MDS patients.

**Methods:** We systematically searched MEDLINE, Embase, Web of Science, and Cochrane Library through March 15, 2021. Randomized controlled trials (RCTs) on AML or HR-MDS patients comparing the efficacy and safety between DAC and AZA or comparing one of HMAs to conventional care regimens (CCR) were selected.

**Results:** Eight RCTs (*n* = 2,184) were identified in the NMA. Four trials compared AZA to CCR, and four compared DAC to CCR. Direct comparisons indicated that, compared to CCR, both AZA and DAC were associated with higher overall response (OR) rate (AZA vs. CCR: relative risk (RR) = 1.48, 95% CI 1.05–2.1; DAC vs. CCR: RR = 2.14, 95% CI 1.21–3.79) and longer overall survival (OS) (AZA vs. CCR: HR = 0.64, 95% CI 0.50–0.82; DAC vs. CCR: HR = 0.84, 95% CI 0.72–0.98), and AZA showed higher rate of complete remission with incomplete blood count recovery (CRi) (HR = 2.52, 95% CI 1.27–5). For the indirect method, DAC showed a higher complete remission (CR) rate than AZA in patients with both AML (RR = 2.28, 95% CI 1.12–4.65) and MDS (RR = 7.57, 95% CI 1.26–45.54). Additionally, DAC significantly increased the risk of 3/4 grade anemia (RR = 1.61, 95% CI: 1.03–2.51), febrile neutropenia (RR = 4.03, 95% CI: 1.41–11.52), and leukopenia (RR = 3.43, 95% CI 1.64–7.16) compared with AZA. No statistical significance was found for the other studied outcomes.

**Conclusion:** Compared to CCR, both AZA and DAC can promote outcomes in patients with AML and HR-MDS. DAC showed higher efficacy especially CR rate than AZA (low-certainty evidence), while AZA experienced lower frequent grade 3/4 cytopenia than patients receiving DAC treatment.

## Introduction

Acute myeloid leukemia (AML) and higher-risk myelodysplastic syndromes (HR-MDS) are heterogeneous hematologic malignancies with clinical manifestations of anemia, hemorrhage, and infection (Arber, 2019). HR-MDS are defined as patients with intermediate-2 or high-risk score by the International Prognostic Scoring System (IPSS) or with intermediate, high, or very high-risk score by the Revised International Prognostic Scoring System (IPSS-R) (Pfeilstocker et al., 2016). HR-MDS are aggressive disorders with rapid progression to AML, with a poor prognosis despite intensive chemotherapy (IC). The annual incidence rates of AML are higher than 4.2 per 100,000 per year (Shallis et al.,2019). The 2- and 5-year overall survival (OS) rates of elderly AML patients are approximately 10 and 2%, respectively (Menzin et al., 2002; Daly and Paquette, 2019). Allogeneic hematopoietic stem cell transplantation (allo-HSCT) is considered to be the only curative treatment for HR-MDS and AML (Stone, 2009). Limited by HLA-matching donor, physical status, ages, costs, treatment-related mortality (TRM), and graft-versus-host disease (GVHD), many patients are ineligible for allo-HSCT. Therefore, it is urgent to develop an effective therapeutic approach for these patients who are ineligible for transplantation.

Azacitidine (AZA) and decitabine (DAC) are lower-intensity chemotherapy agents and have been approved to treat MDS by the Food and Drug Administration (FDA). On September 1, 2020, FDA approved oral AZA for the maintenance treatment of patients with AML. Hypomethylating agents (HMAs) have become the standard therapy for patients with HR-MDS or AML who are not candidates for allo-HSCT and intensive chemotherapies (Sanz, 2019). These two agents are slightly different in structure: AZA is a ribonucleoside, while DAC is a deoxyribonucleoside (Lyko and Brown, 2005). Both AZA and DAC act by depletion of DNA methyltransferases. However, these two agents have different mechanisms of action: 80–90% of AZA is integrated into RNA, leading to abnormal ribosome assembly and inhibiting tumor-related protein synthesis; 10%–20% can also be converted into 5-aza-2'-deoxycytidine by the action of ribonucleotide reductase to bind to DNA, thereby inhibiting DNA methyltransferase and leading to the reexpression of tumor suppressor genes. While DAC is incorporated only into DNA, high-dose DAC inhibits DNA cross-linking and synthesis through cytotoxicity, and low-dose DAC exerts DNA demethylation by inhibiting DNA methyltransferase, reactivating silent tumor suppressor genes (Stresemann and Lyko, 2008; Hollenbach et al., 2010). Preclinical studies have shown that DAC is more effective than AZA in antileukemia activity *in vivo* (Cany et al., 2018); however, clinical data indicate that AZA is more effective than DAC. Observational studies of these two agents have shown similar efficacy and toxicity profiles in the treatment of refractory anemia with excessive blasts (MDS-RAEB) (Salim et al., 2016). Compared with CCR, both AZA and DAC have shown delayed progression to AML (Fenaux et al., 2009; Kantarjian et al., 2006; Lubbert et al., 2011; Silverman et al., 2002). However, only AZA has shown a significant advantage in OS compared with CCR (median OS, 24.5 vs. 15 months, respectively) in patients with HR-MDS and AML with 20–30% marrow blasts (Fenaux et al., 2009), establishing it as the first-line treatment of choice for those patients who are unfit for transplant (Santini et al., 2010).

Up to now, direct comparison of AZA and DAC has been performed in rare randomized trials, leading to the dilemma choice of these two agents for patients and physicians. Several meta-analyses have been conducted to compare the efficacy and safety of AZA and DAC in MDS or AML patients. None of them made a comparison in HR-MDS and AML. Therefore, the objective of this study was to compare the efficacy, safety, and survival of AZA and DAC in patients with HR-MDS and AML.

## Methods

We prospectively registered the current review in the PROSPERO database (registration number: CRD42021245905). The Preferred Reporting Items for a Systematic Review and Meta-Analysis of Diagnostic Test Accuracy (PRISMA-DTA) studies guideline was followed in preparing this systematic review.

### Search Strategy

We systematically searched all studies published in MEDLINE (*via* PubMed), Web of Science, Cochrane Library, and Embase through March 15, 2021, without time or language restrictions. Keywords included “hypomethylating agents”, “azacitidine”, “decitabine”, “myelodysplastic syndrome”, and “acute myeloid leukopenia”. The detailed search strategies were listed in Supplementary Table S1.

### Study Selection, Inclusion, and Exclusion Criteria

All randomized controlled trials (RCTs) comparing HMAs to CCR (including best supportive care (BSC), low-dose Ara-C (LDA), and IC) or AZA to DAC in patients with HR-MDS and AML were included in this study, regardless of publication status and language. Reviews, case reports, meta-analyses, and preclinical and observational studies were excluded. Two reviewers (Jiale Ma and Zheng Ge) screened all references identified through our search and inclusion criteria. Disagreements were settled by discussion of the two reviewers and involved a third independent reviewer if necessary.

Phases II and III and RCTs were selected in this systematic review and network meta-analysis (NMA). Adult patients diagnosed with AML and/or MDS were selected. The treatment options included single agent AZA or DAC, or comparison of these two drugs against each other, or comparison of them to CCR without previous allo-HSCT or other chemotherapies. Of note, in MDS studies, the included population of higher-risk MDS should be more than 60% of all MDS patients. In other words, the included population should be mainly HR-MDS. Additionally, at least one of the relevant outcomes should be reported in the trial including objective remission (OR), complete remission (CR), complete remission with incomplete blood count recovery (CRi), complete remission with incomplete platelet recovery (CRp), partial response (PR), hematological improvement (HI), marrow complete remission (mCR) rates, or AEs or at least one form of survival data.

Exclusion criteria included patients with therapy-related disease; prior treatment with AZA, DAC, chemotherapy, immunotherapy, or planned allo-HSCT.

### Date Extraction and Clinical Endpoint

Extracted data included 1) study characteristics (author, publication year, and study type); 2) patient characteristics (age, gender, WHO/FAB classification, disease stage using IPSS criteria, karyotype risk, and ECOG score); 3) the hypomethylating treatment regimen; 4) the outcome measures [CR, CRi, CRp, PR, HI, mCR, overall response (OR) rates, drug-related AEs rate, and OS]. The primary outcomes were efficacy (response rate measured by a total number of included patients) and AEs. The second outcomes were OS of all patients. In the absence of information or supplemental data from the authors, the response rate was calculated according to a validated imputation method (Furukawa et al., 2005).

### Quality Assessment

Cochrane Handbook for Systematic Reviews of Interventions was used to assess the bias of each included RCT. The criteria for evaluation included random sequence generation, allocation concealment, blinding, incomplete outcome data, selective outcome reporting, and other sources of bias. The risk of bias was assessed as low, unclear, or high.

### Statistical Analysis

All the NMAs were performed by using the meta-analysis program of STATA 14.0 software (Stata Corporation, Texas) and Review Manager 5.4 software (Cochrane Collaboration, Oxford, United Kingdom). Direct pairwise meta-analyses were first performed to estimate the available relative effects of the competing interventions using the random effects model. The binomial distribution was used to calculate and express relative risks (RRs) and 95% confidence intervals (95% CIs). Heterogeneity parameters (I^2^) for each pairwise comparison were quantified to express a percentage of variability, and that is due to true differences between studies rather than sampling error (Higgins and Thompson, 2002). All analyses were performed by using the Mantel–Haenszel (M–H) method.

We performed an NMA to analyze all comparisons among interventions for each outcome. This is because NMA takes advantage of two statistical approaches. First, the use of indirect comparisons can help us to estimate the effect of intervention A versus intervention B, indirectly if both A and B have been compared against an intervention C. Second, the combination of direct and indirect comparisons allows reviewers to obtain more precise estimates (Nino-Serna et al., 2020). In the presence of both direct and indirect evidence, the NMA provided a combined effect estimate. A random effects model of NMA was conducted for each outcome using the multivariate meta-analysis approach. For each outcome and a connected network of studies, we performed a frequentist framework NMA if the assumptions of between-study homogeneity, transitivity, and consistency of evidence across treatment comparisons were judged to be justifiable (Baker and Kramer, 2002; Cipriani et al., 2013). Inconsistency network models were used to test the global consistency of direct and indirect estimates for pairwise comparisons, and node-splitting method models were used to test the local inconsistency. Design-by-treatment interaction models (Higgins et al., 2012) were used to statistically evaluate the consistency. We assume that the treatment comparisons have common heterogeneity because the included treatments have the same properties and sharing common heterogeneity parameters is clinically reasonable. The graph and summary of risk of bias were created to assess the bias within studies. Surface under the cumulative ranking (SUCRA) (Salanti et al., 2011) probabilities were used to rank the treatment for the outcome. For patients with HR-MDS or AML, larger SUCRA values indicate higher rank of the treatment. In addition, a clustered ranking plot was constructed using SUCRA values for efficacy and safety outcomes to obtain information on meaningful groups of treatments that maximize benefits for efficacy and safety outcomes.

## Results

### Literature Search Results

**FIGURE 1:**
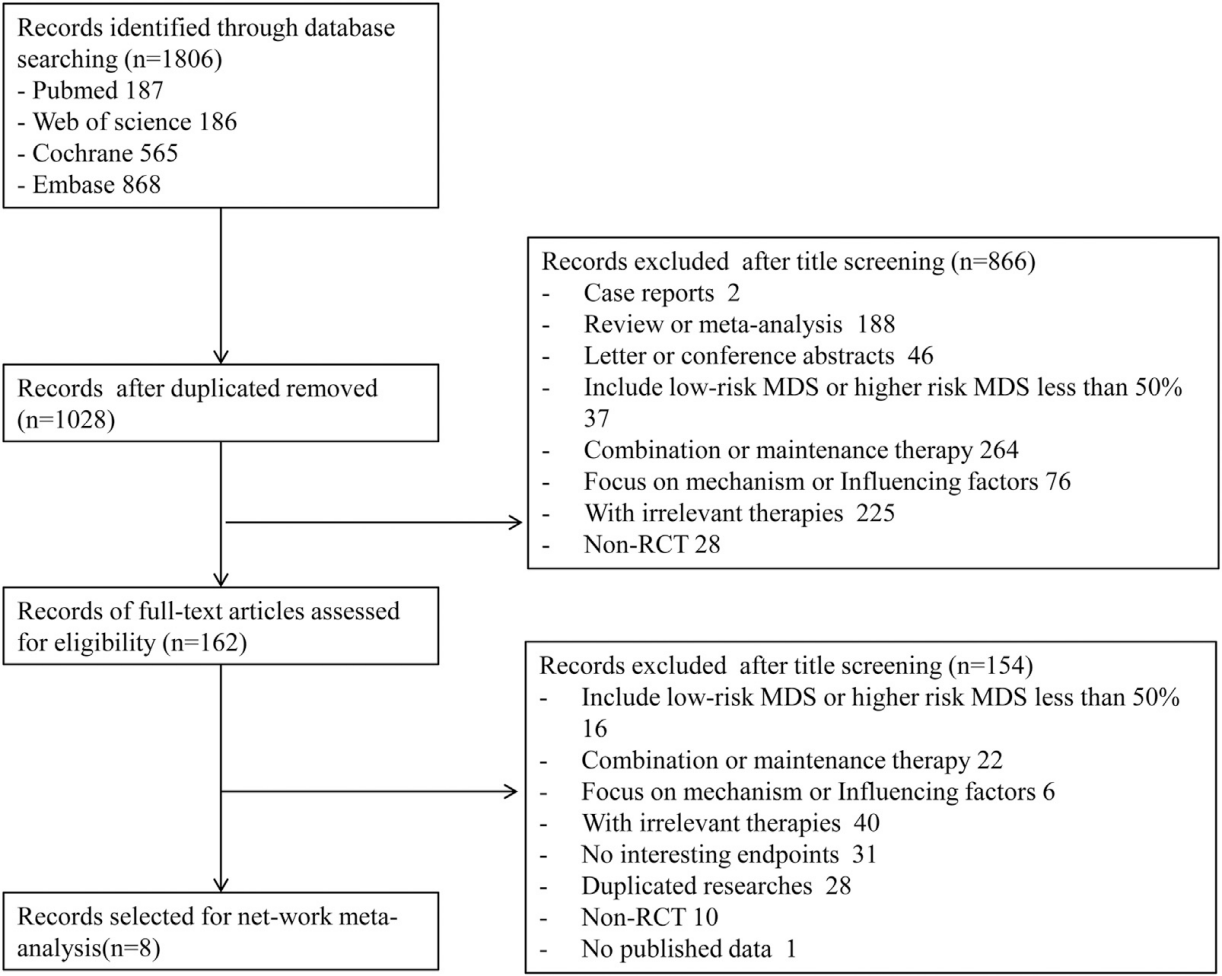
Flowchart presenting the steps of the literature search and selection.

**FIGURE 2:**
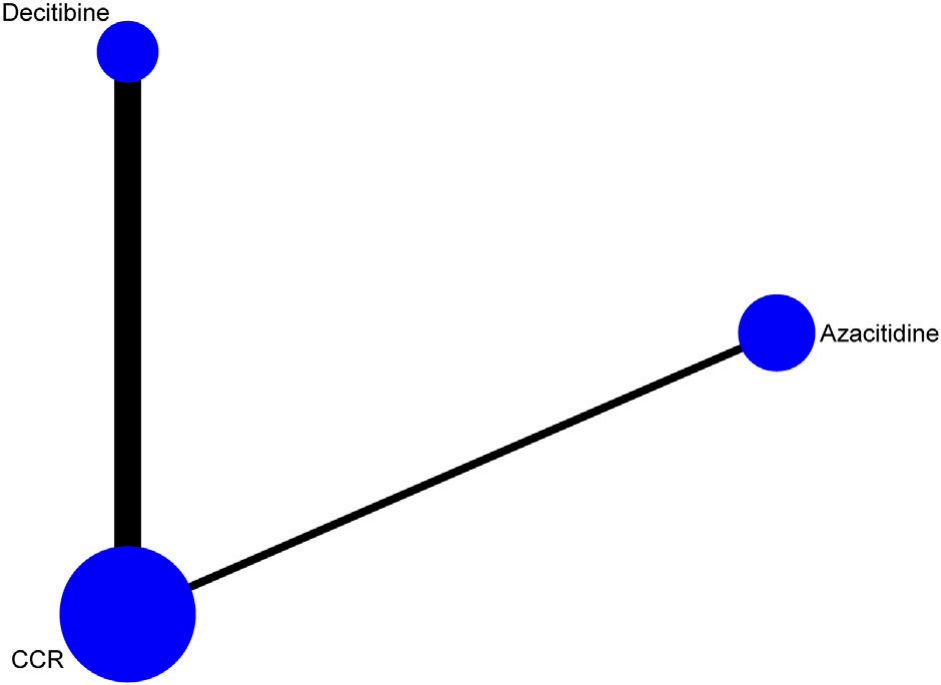
Network of interventional treatments comparing types of acute myeloid leukemia and higher-risk myelodysplastic syndrome (CR). The sizes of the nodes represent the total sample size for each treatment. Line thickness and the numbers beside the lines correspond to the number of trials. CCR, conventional care regimens.

A total of 1,806 records were obtained with the search strategy. After removing 778 duplicates, 1,028 records were screened by title and abstract. A total of 866 records were excluded due to ineligibility. 162 records of full-text articles were assessed for eligibility. 154 records were excluded after screening full-text articles. Of note, the results of Becker et al. (2015)’s study in 2015 were a subgroup analysis of the randomized phase III study 06,011 of the EORTC Leukemia Cooperative Group and German MDS Study Group (GMDSSG) (Lubbert et al., 2011). Despite being a same study, these two articles focused on different aspects. Lubbert et al.’s study involved all risk-stratified MDS patients, while Becker et al.’s study included only RAEB-t patients, which was more representative in the high-risk group. If we only include Becker et al.’s study, the other middle- and high-risk patients of the entire experimental group will be ignored. After weighing it, repeatedly, we included both studies in the statistical analysis, although it may bring selected offsets. Finally, eight trials were eligible for extraction for this NMA (Figure 1). As indicated in the network plot (Figure 2), AZA vs. CCR and DAC vs. CCR are the most prevalent comparisons.

### Publication Characteristics

The characteristics of publication were listed in Table 1. Eight RCTs involved 2,184 patients with a median age of 71.1 years (IQR 68.4–73.8). Four RCTs involved a number of 1,221 patients compared to AZA (75 mg/m2/day for 7 days every 28-day cycle for at least six cycles) and the CCR, including BSC, LDA, and I. Four RCTs involving 963 patients compared DAC (15–20 mg/m2/day for 3–5 days every 4 28-day cycles) to CCR. Among the eight RCTs, four were about the application of HMA in MDS, and four were in AML. According to IPSS scores, more than 70% of patients had intermediate-2 or high-risk MDS (Becker et al., 2015; Fenaux et al., 2009; Kantarjian et al., 2006; Lubbert et al., 2011; Inc E et al., 2014). The Eastern Cooperative Oncology Group (ECOG) performance status scores of patients from seven trials are between 0 and 2. For response data, all trials with MDS applied International Working Group (IWG) 2,000 response criteria (Cheson et al., 2000), and all trials with AML applied IWG 2004 response criteria (Creutzig and Kaspers, 2004). AEs were assessed with the National Cancer Institute’s Common Toxicity Criteria, version 2.0/3.0. Grade 3–4 AEs are the main research (http://ctep.cancergov/reporting/ctc_archive.html).

**TABLE 1:** Characteristics of publications (Supplementary Material).

Study, year	Type	Intervention (dose, schedule)	Patients enrolled	Female	Age, median	WHO classification	FAB classification	IPSS	Karyotape risk	ECOG	Median cycles	Efficacy	Grade3/4) adverse events	Median OS (months)
Fenaux et al. (2009)	Phase III	Aza (75 mg/m2/d*7d per 28-day cycle for at least 6 cycles)	179	47	69 (42–83)	RAEB-1 14, RAEB-2 98, CMML-1 1, CMML-2 10, AML 55, Indterminate 1	RAEB 104, RAEB-T 61, CMML 6, AML 1	IPSS-1 5, IPSS-2 76, High 82	Favorable 83, Intermediate 37, Unfavorable 50, unknown 9	0: 78,1: 86, ≥2: 13, unknown: 2	9 (4–15)	OR 138, CR 30, PR 21, HI 87	Neutropenia 159, thrombocytopenia 149, anaemia 100,death 82	24.5
		CCR(BSC 105, LDA49, intensivechemotherapy 25)	179	60	70 (38–88)	RAEB-1 17, RAEB-2 95, CMML-1 0, CMML-2 5, AML 58, Indterminate 4	RAEB 103, RAEB-T 62, CMML 5, AML 1	IPSS-1 13, IPSS-2 70, High 85	Favorable 84, Intermediate 39, Unfavorable 50, unknown 6	0: 80,1: 86, ≥2: 10, unknown: 3	NA	OR 72, CR 14, PR 7, HI 51	Neutropenia 126, thrombocytopenia 132, anaemia 112, death 113	15
Dombret et al. (2015)	Phase III	Aza(75 mg/m2/d*7d per 28-day cycle for at least 6 cycles)	241	102	75(64–91)	AML	AML	−	−		6(1–28)	OR 75, Cri 20, CR 47, PR 3	Febrile neutropenia 66, pneumonia 45, leukopenia 16, hypokalemia 12, neutropenia 62, thrombocytopenia 56, anaemia 37, death 193	10.4
		CCR (BSC, LDAC(20 mg bid *10d per 28-day treatment cycle for at least 4 cycles),IC (Ara-c100-200 mg/m2/ d *7d, daunorubicin 45-60 mg/m2/d*3d or idarubicin 9-12 mg/m2/d*3d)	247	98	75(65–89)	AML	AML	−	−	−	2 (1-3) IC cycles, and 4 (1-25) LDAC cycles, and the median exposure to BSC only was 65 (6-535) days	OR 65, Cri 8,CR 54 , PR 3	Febrile neutropenia 70, pneumonia 33, leukopenia 19, hypokalemia 18, neutropenia 54, thrombocytopenia 53, anaemia 43, death 201	6.5
Kantarjian et al. (2012)	Phase III	Dec (20 mg/m(2)/d*5d per 28-day cycle)	242	105	73(64–89)	AML	AML	−	Favorable NA, Intermediate 152, Unfavorable 87, unknown NA	1: 184, ≥2: 58	NA	OR 49, Cri 24, CR 38, CRp 5, PR 6	Febrile neutropenia 76, pneumonia 51, leukopenia 47, hypokalemia 27, neutropenia 76, thrombocytopenia 95, anaemia 80,dyspnea 16, death 197	7.7
		CCR (BSC or cytarabine 20 mg/m(2)/d as a subcutaneous injection for 10 consecutive days every 4 weeks)	243	92	73(64–91)	AML	AML	−	Favorable NA, Intermediate 154, Unfavorable 87, unknown NA	1: 183, ≥2: 60	NA	OR 28, Cri 7, CR 18, CRp 1, PR 9	Febrile neutropenia 51, pneumonia 43, leukopenia 20, hypokalemia 24, neutropenia 42, thrombocytopenia 77,anaemia 60,dyspnea 14, death 199	5
Lübbert et al. (2011)	Phase II	Dec (15 mg/m2 q8h *3 d,every 6-week cycles)	119	43	69(60–90)	MDS	RAEB 61,RAEB-T 40,CMML 10, AML 1	IPSS-1 8, IPSS-2 64, High 46, unknown 1	Favorable 38, Intermediate 8, Unfavorable 57, unknown 15	0: 29,1: 76, ≥2: 14,	4	OR 41,CR 16,PR 7, HI 18	Febrile neutropenia 29, pneumonia 66, neutropenia 54, thrombocytopenia 20,anaemia NA,death 99	10.1
		BSC	114	41	70(60–86)	MDS	RAEB 64,RAEB-T 35,CMML 4, AML 1	IPSS-1 8, IPSS-2 63, High 42, unknown 1	Favorable 29, Intermediate 17, Unfavorable 51, unknown 17	0: 25,1: 72, ≥2: 17	NA	OR 28, Cri 7, CR 18, CRp 1, PR 9	Febrile neutropenia 8, pneumonia 57, neutropenia 40, thrombocytopenia 18,anaemia NA, death 96	8.5
Seymour et al. (2017)	RCT	Aza (75 mg/m2/d*7d per 28-day cycle)	129	48	76 (64–90)	AML	AML	−	Favorable NA, Intermediate 63, Unfavorable 66,	1: 94, ≥2: 35	5(1–27)	OR 33, Cri 7, CR 25, PR 1	Febrile neutropenia 29, pneumonia 24, leukopenia 8, hypokalemia 9,neutropenia 28, thrombocytopenia 33,anaemia 19,dyspnea 6, sepsis 7, death NA	8.9
		CCR	133	55	75 (65–87)	AML	AML	−	Favorable NA, Intermediate 61, Unfavorable 72,	1: 104, ≥2: 29	2	OR 25, Cri 3, CR 20, PR 2	Febrile neutropenia 43, pneumonia 18,, leukopenia 10, hypokalemia 10, neutropenia 25, thrombocytopenia 27,anaemia 21,dyspnea 4, sepsis 9, death NA	4.9
Fenaux et al. (2010)	Phase III	Aza (75 mg/m2/d*7d per 28-day cycle for at least 6 cycles)	55	18	70(52–80	AML	AML	−	Favorable 19, Intermediate 38, Unfavorable 14, unknown 3	0: 16,1: 35, ≥2: 4, unknown 0	NA	CR 10	Neutropenia 50, thrombocytopenia 48,anaemia 30,death NA	24.5
		CCR	58	17	70(50–83)	AML	AML	−	Favorable 33, Intermediate 43, Unfavorable 13, unknown 2	0: 22,1: 34, ≥2: 0, unknown 2	NA	CR 9	Neutropenia 44, thrombocytopenia 44, anaemia 36, death NA	16
Kantarjian et al. (2006)	Phase III	Dec(15mg/m2 q8h*3d,every 6 weeks)	89	30	70(65–76)	NA	RA 12, RARS 7, RAEB 47, RAEB-T 17, CMML 6	IPSS-1 28, IPSS-2 38, High 23	NA	0: 21,1: 61, ≥2: 4, unknown 0	3(0–9)	OR 27, CR 8, PR 7, HI 12	Febrile neutropenia 23, pneumonia 15, leukopenia 22, neutropenia 87, thrombocytopenia 85, anaemia 12, death 12	14
		BSC	81	24	70(62–74)	NA	RA 12, RARS 4, RAEB 43, RAEB-T 14, CMML 8	IPSS-1 24, IPSS-2 36, High 21	NA	0: 28,1: 48, ≥2: 4, unknown 1	NA	OR 6,HI 6	Febrile neutropenia 4, pneumonia 9, leukopenia 7,neutropenia 50, thrombocytopenia 43, anaemia 15, death 18	14.9
Becker et al. (2015)	Phase III	Dec (15 mg/m2 q8h *3 d,every 6-week cycles)	40	11	69.5(61–90)	MDS	RAEB-t	IPSS-1 2, IPSS-2 12, High 26	Favorable 16, Intermediate 4, Unfavorable 14, unknown 6	0: 8,1: 29, ≥2: 3	NA	OR 12, CR 4, PR 2, HI 6	NA	8
		BSC	35	11	69(61–80)	MDS	RAEB-t	IPSS-1 0, IPSS-2 13, High 22		0: 10,1: 19, ≥2: 6	NA	OR 0, CR 0, PR 0, HI 0	NA	6

AZA, azacitidine; DAC, decitabine; CCR, conventional care regimens (including best supportive care, low-dose cytarabine, and intensive chemotherapy); BSC, best supportive care; RAEB, refractory anemia with excess blasts; RAEB-T, refractory anemia with excess blasts transformation; NA, not available; OR, objective remission; CR, complete remission, CRi, complete remission with incomplete blood count recovery; CRp, complete remission with incomplete platelet recovery; PR, partial response; HI, hematological improvement; mCR, marrow complete remission.

### Risk of Bias

**FIGURE 3:**
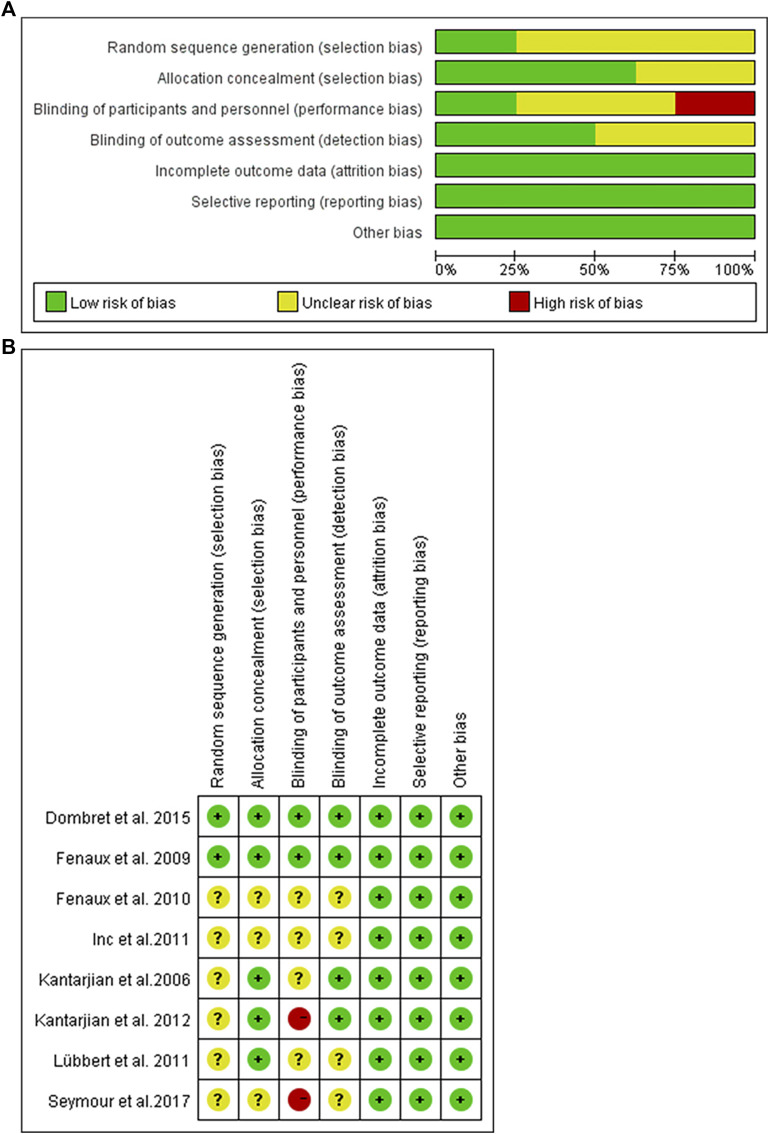
Risk of bias graph. **(A)** Authors’ judgments about each risk of bias item presented as percentages across all included studies. **(B)** Risk of bias summary. Authors’ judgments about each risk of bias item for each included study.

The risk of bias among studies ranges from low and unclear to high. Random sequence generation was adequate in two trials, whereas allocation concealment was achieved in six trials and blinding of outcome assessor in two trials. In addition, selective reporting and incomplete outcome data were low risk in all trials. The graph and summary of the risk of bias are shown in Figure 3.

### Assessment of Inconsistency

Inconsistency tests between direct and indirect estimates in AZA versus DAC were nonsignificant (*p* > 0.05), indicating that indirect estimates were not different to direct evidence. The estimation of NMA inconsistency between AZA and DAC was listed in Supplementary Table S2. The overall level of each treatment met the consistency assumption (*p* > 0.05).

### Results of NMA

#### Comparison of Efficacy Between Decitabine and Azacitidine

**FIGURE 4:**
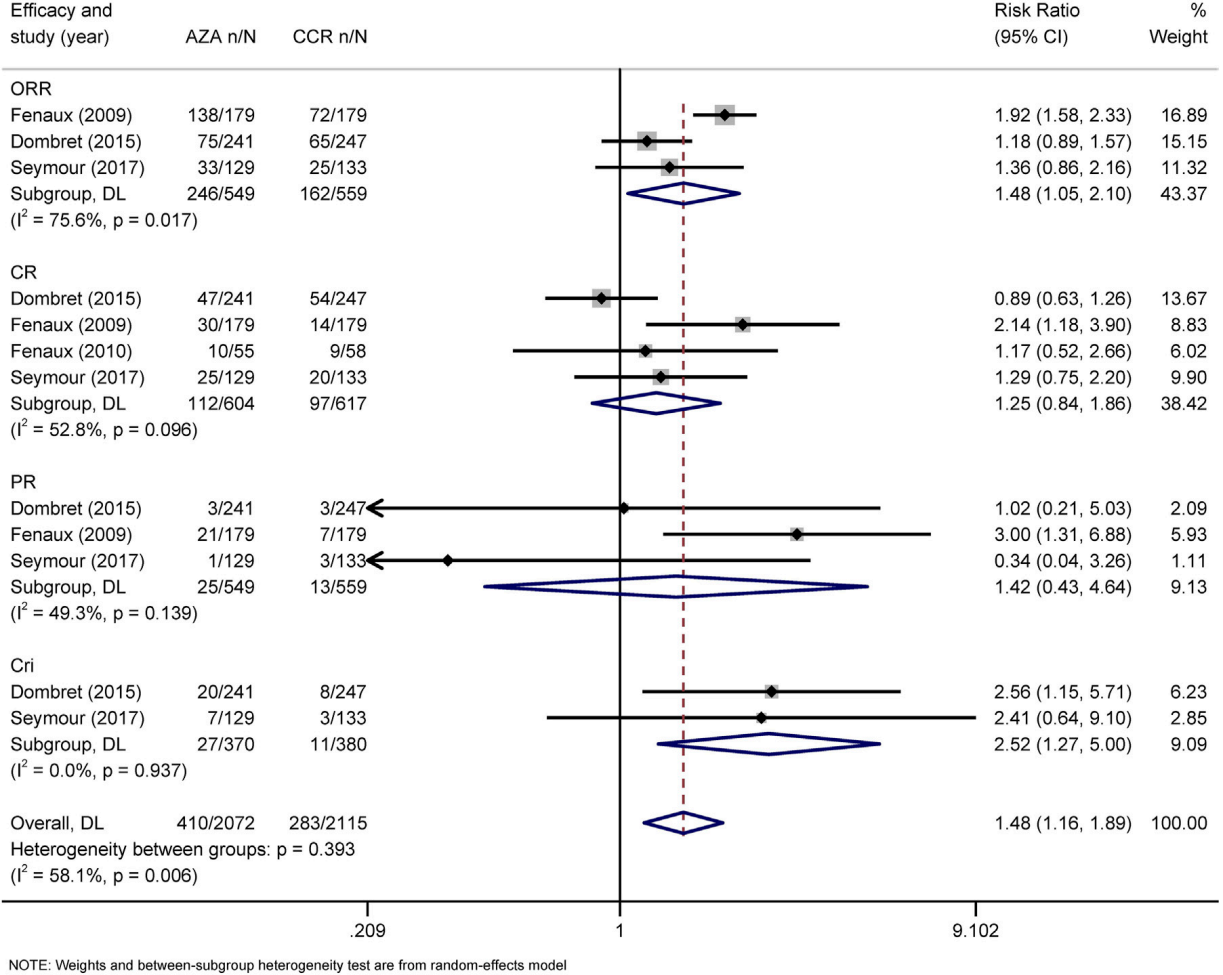
Forest plot of efficacy of azacitidine vs. conventional care regimens (direct evidence-RR). Forest plot represents the direct comparison of efficacy between AZA and CCR. RR, relative risks; 95%CIs, 95% confidence intervals; CCR, conventional care regimens; n, total number of events; N, total number of patients.

**FIGURE 5:**
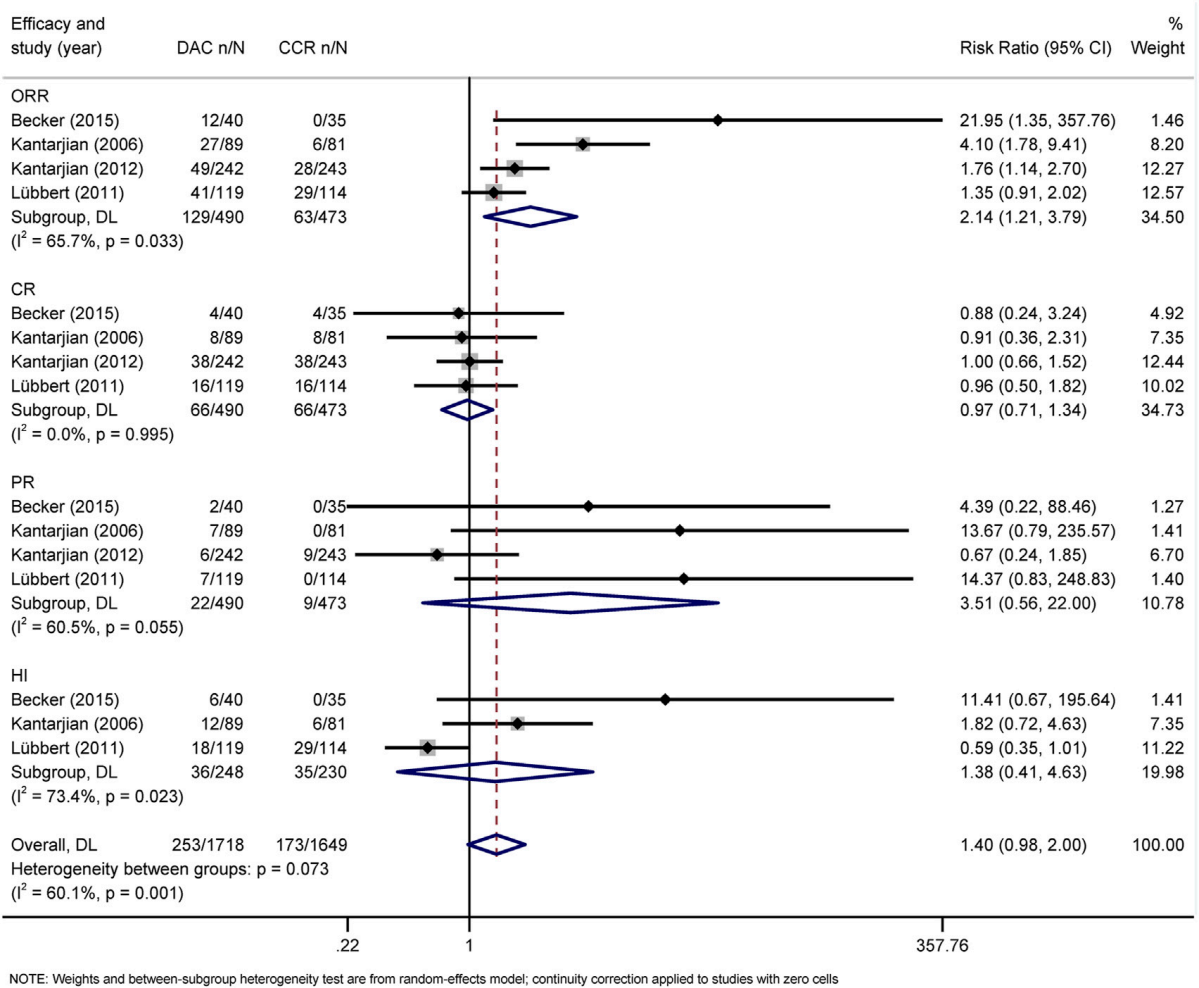
Forest plot of efficacy of decitabine vs. conventional care regimens (direct evidence-RR).

The primary efficacy endpoints were OR, CR, PR, CRi, and HI rates. IWG 2000 response criteria were used in all patients with MDS, while IWG2003 response criteria were applied in patients with AML. Direct comparison of HMAs with CCR showed that AZA significantly increased the rates of OR *(*RR = 1.48, 95% CI 1.05–2.1) and CRi (HR = 2.52, 95% CI 1.27–5) (Figure 4), while DAC only increased the rate of OR (RR = 2.14, 95% CI 1.21–3.79) (Figure 5). Concerned about the high heterogeneity (*I* ^*2*^ > 50%), a subgroup analysis by disease type was estimated. In AML, AZA showed a higher CRi rate than CCR (RR = 2.52, 95% CI 1.27–5.00) (Supplementary Figure S1). In MDS, DAC significantly increased the PR rate than CCR (RR = 9.78, 95% CI 1.83–52.09) (Supplementary Figure S4). There were no statistically significant differences in other outcomes.

**FIGURE 6:**
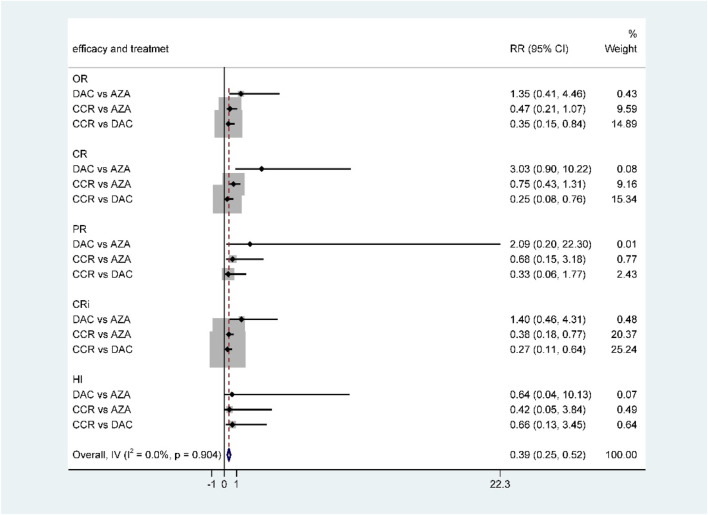
Forest plot of efficacy represents the direct and indirect comparison.

For NMA, there were no statistically significant differences in terms of OR, CR, PR, CRi, and HI between DAC and AZA (Figure 6; Table 2). However, when performing subgroup analysis by disease type, DAC showed a higher CR rate than AZA both in AML (RR = 2.28, 95% CI 1.12–4.65) (Supplementary Figure S3) and in MDS (RR = 7.57, 95% CI 1.26–45.54) (Supplementary Figure S6).

**TABLE 2:** Summary of the SUCRA of efficacy and high-grade side effects.

Outcome and data	AZA	DAC	CCR	*p*-value of the design-by-treatment test
Overall response rate	63.7	84.3	2.1	0.625
Complete remission	44.1	97.6	8.3	0.074
Partial remission	47.4	82.1	20.6	0.54
Complete remission with incomplete blood count recovery	6.39	85.9	0.2	0.553
Hematology improvement	64.7	55.4	29.8	0.876
Neutropenia	49.9	0.1	100	0.005
Thrombocytopenia	47.8	13	89.2	0.434
Anemia	96	4.9	49.1	0.037
Febrile neutropenia	97.1	0.3	62.7	0.009
Pneumonia	16.5	37.6	91.9	0.589
Leukopenia	86.6	0	63.4	0.002
Hypokalemia	82.8	24.7	42.5	0.324
Death	83	45.2	21.8	0.489

SUCRA, surface under the cumulative ranking curve.

#### Comparison of Grade 3/4 Adverse Events (HTEs) Between Decitabine and Azacitidine

**FIGURE 7:**
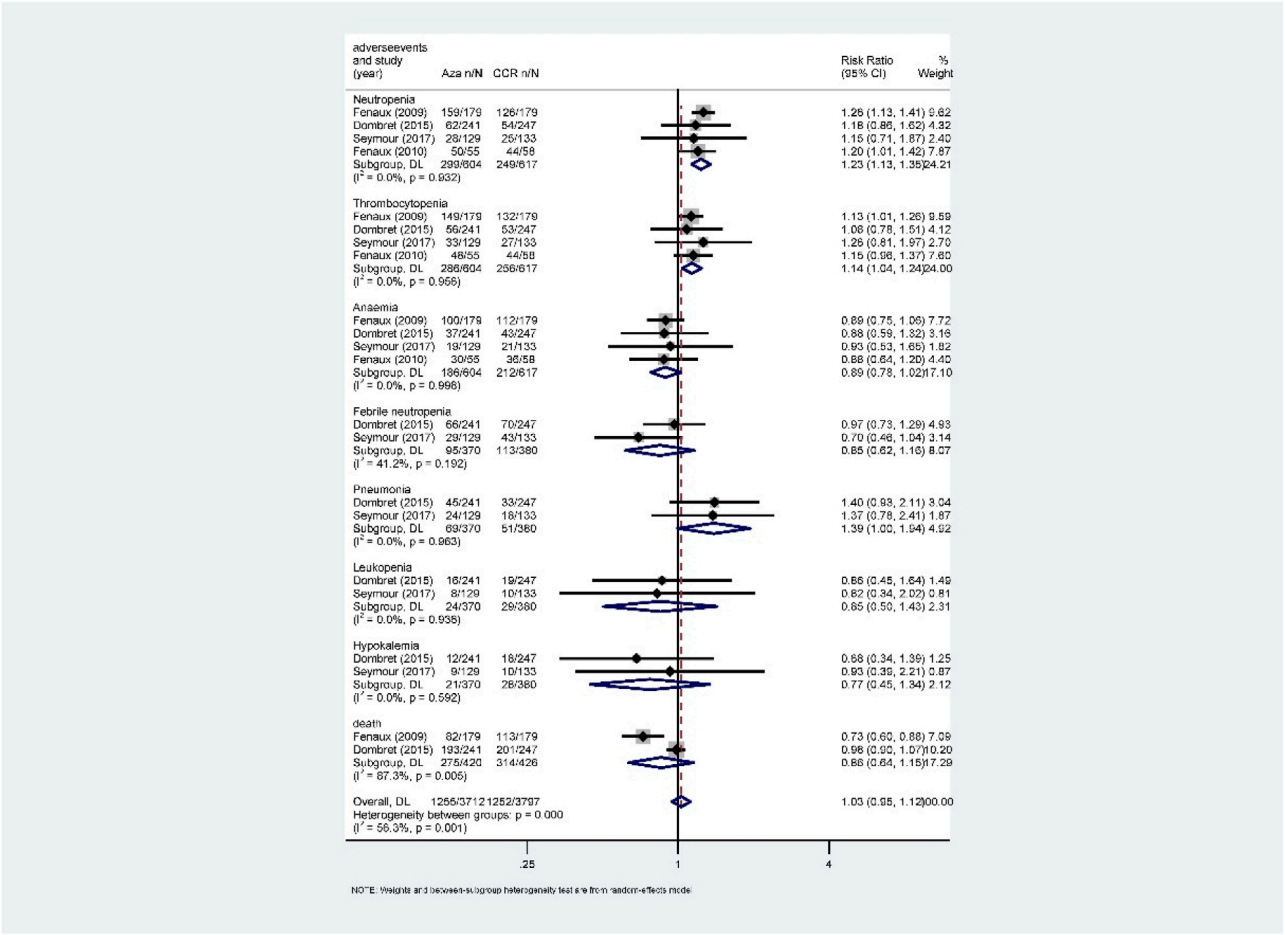
Forest plot of grade 3/4 adverse events of azacitidine vs. conventional care regimens (direct evidence-RR).

**FIGURE 8:**
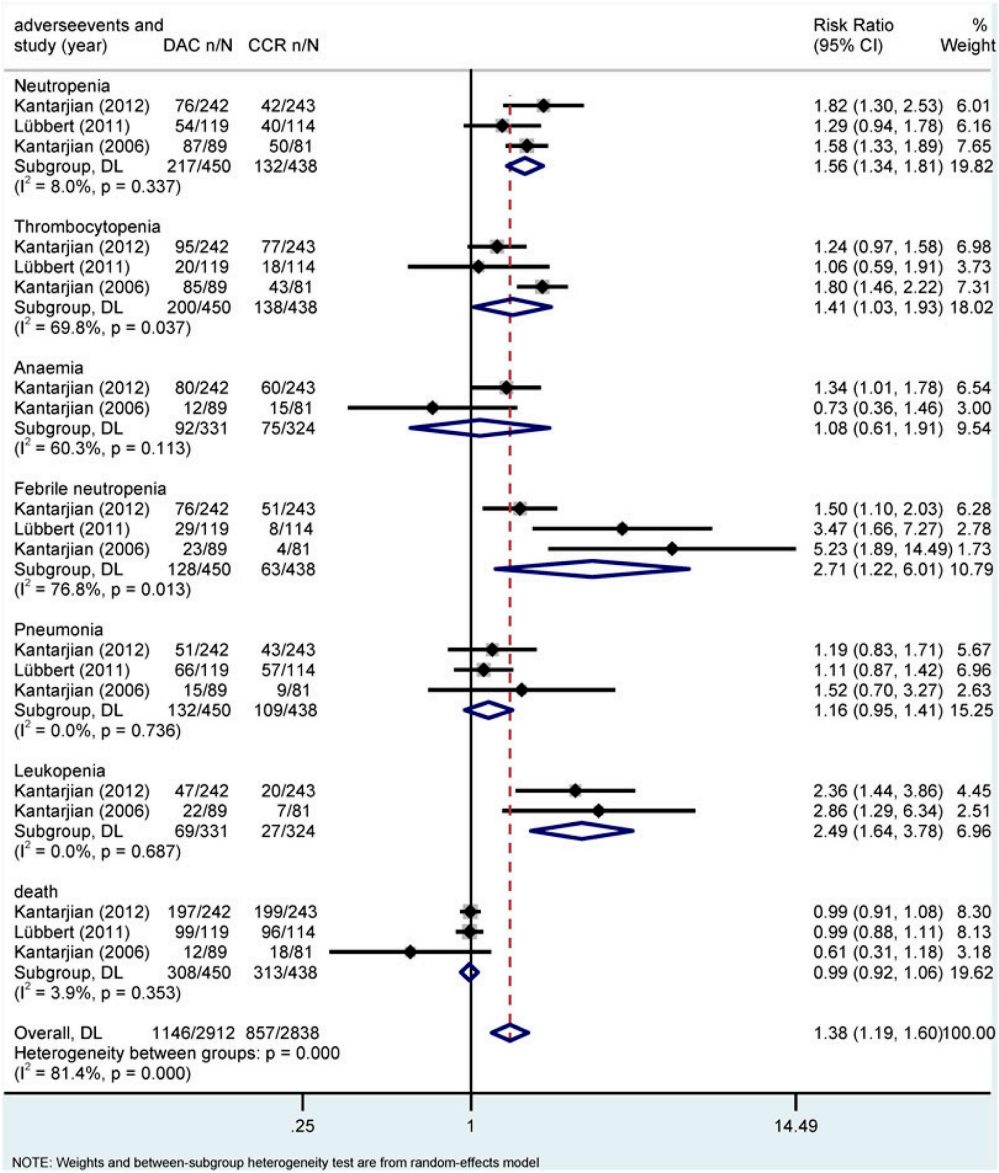
Forest plot of grade 3/4 adverse events of decitabine vs. conventional care regimens (direct evidence-RR).

Hematological toxicity was the most common adverse event in HMA treatment, which included leukopenia, neutropenia, thrombocytopenia, anemia, and febrile neutropenia. Additionally, nonhematological adverse reactions such as pneumonia and hypokalemia also occurred in patients with HR-MDS or AML. In this study, a total of 2,135 patients from eight studies who received HMAs were included for analysis (Dombret et al., 2015; Fenaux et al., 2009; Fenaux et al., 2010; Kantarjian et al., 2006; Kantarjian et al., 2012; Lubbert et al., 2011; Seymour et al., 2017; Inc E et al., 2014). Subgroup of direct comparisons showed that, compared to CCR, AZA significantly increased the risk of grade 3/4 neutropenia (RR = 1.23, 95% CI: 1.13–1.35) and thrombocytopenia (RR = 1.14, 95% CI: 1.04–1.24) (Figure 7), and DAC increased the risk of grade 3/4 neutropenia (RR = 1.56, 95% CI: 1.34–1.81), thrombocytopenia (RR = 1.41, 95% CI: 1.03–1.93), febrile neutropenia (RR = 2.71, 95% CI: 1.22–6.01), and leukopenia (RR = 2.49, 95% CI: 1.64–3.78) (Figure 8). In AML, AZA significantly increased the risk of grade 3/4 neutropenia (RR = 1.19, 95% CI: 1.03–1.37) (Supplementary Figure S2). In MDS, DAC increased the risk of grade 3/4 neutropenia (RR = 1.50, 95% CI: 1.25–1.79), febrile neutropenia (RR = 4.00, 95% CI: 2.2–7.28), and leukopenia (RR = 2.86, 95% CI: 1.29–6.34) (Supplementary Figure S5). There was no statistically significant difference found in other studied outcomes.

**FIGURE 9:**
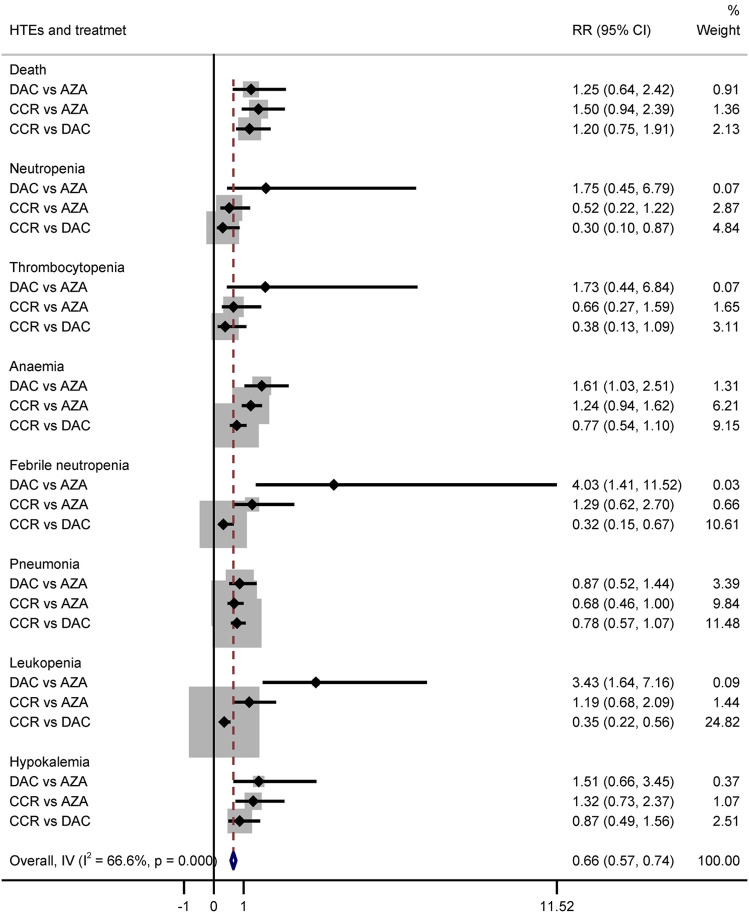
Forest plot of grade 3/4 adverse events represents the direct and indirect comparison.

The results of the indirect comparison of AZA and DAC showed that DAC significantly increased the risk of high-grade anemia (RR = 1.61, 95% CI: 1.03–2.51), febrile neutropenia (RR = 4.03, 95% CI: 1.41–11.52), and leukopenia (RR = 3.43, 95% CI: 1.64–7.16) compared with AZA (Figure 9). The results were the same in patients with AML (Supplementary Figure S3). There was no statistical significance in the association of other HTEs in groups treated with DAC compared with AZA.

#### Comparison of Survival Between Decitabine and Azacitidine

**FIGURE 10:**
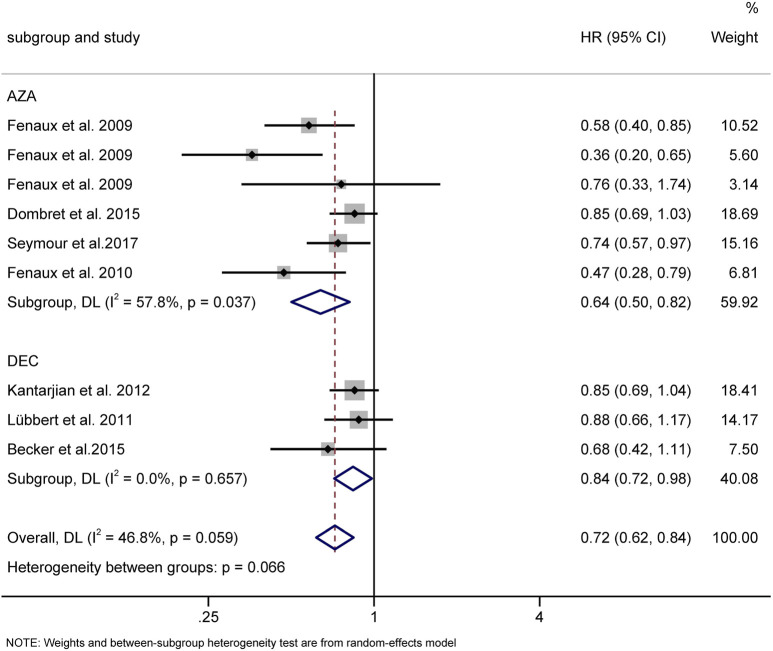
Overall survival of AZA and DAC compared to CCR. AZA, azacitidine; Dec, decitabine; CCR, conventional care regimens.

Seven RCTs were available for the analysis of median OS for HMAs vs. CCR (Becker et al., 2015; Dombret et al., 2015; Fenaux et al., 2009; Fenaux et al., 2010; Kantarjian et al., 2012; Lubbert et al., 2011; Seymour et al., 2017). Compared with CCR, both AZA (HR = 0.64, 95% CI 0.50–0.82) and DAC (HR = 0.84, 95% CI 0.72–0.98) prolonged OS (Figure 10)

## Discussion

As common HMAs, AZA and DAC are widely used in clinical setting. Both of them have similar clinical effects. However, the clinical choice between them is controversial. In this systematic review and NMA, we aimed to evaluate the comparative efficacy and AEs of AZA and DAC in patients with HR-MDS and AML. In the direct comparisons of HMAs and CCR, we have demonstrated that both AZA and DAC are likely to have better outcomes compared to conventional care regimens (CCR) (including BSC, LDA, and IC) in terms of efficacy and OS. NMA comparisons between AZA and DAC showed that there were no statistically significant differences in efficacy, while the efficacy sorting showed that DAC demonstrated a higher CR rate than AZA in patients with both AML and MDS. Overall, it seems that there is no superiority of one agent over the other in terms of response rates. However, with regard to the safety profile, patients receiving DAC experienced more frequent grade 3/4 cytopenia especially anemia, febrile neutropenia, and leukopenia than patients receiving AZA treatment.

A previous systematic review and NMA published in 2018 compared both HMAs agents to CCR in patients with MDS and has identified four trials. The results showed that HMAs overall improved survival and time to transformation or death (Almasri et al., 2018). Zhang et al. (2021) recently reported a meta-analysis of HMAs for elderly patients with AML. The results showed that HMAs improved the OS and CR rate compared with CCR and also increased the incidence of neutropenia, thrombocytopenia, and pneumonia. Another recent systematic review and NMA identified 1,086 elderly patients with AML from three RCTs to indirectly compare the efficacy and safety of DAC and AZA. The direct comparisons results showed that AZA significantly reduced mortality, while DAC was not compared to CCR. The indirect head-to-head comparisons showed that AZA significantly reduced the mortality rate and anemia. Patients treated with AZA were more likely to achieve CR compared to DAC (Wen et al., 2020). Liu et al. (2021) recently published an NMA which identified six RCTs with 1,072 MDS patients and three RCTs with 1,256 AML patients treated with HMAs. The results showed that, in MDS, AZA showed better AML-free survival, whereas DAC demonstrated higher CR and ORR, and AZA obtained better OS with lower toxicity. In AML, DAC had the possibility of achieving superior CR, ORR, and OS, while the toxicity was relatively higher. Taking these results together, all of the direct comparisons between HMAs and CCR are consistent with our findings. However, for the indirect comparisons of AZA and DAC, both of Almasri et al. (2018) and Wen et al. (2020)’s NMAs showed that AZA was more likely to improve CR compared to DAC, despite being with low-certainty evidence. This was different from our analysis. Our study showed that DAC had the possibility of achieving superior OR, CR, PR, and CRi than AZA, but there were no statistically significant differences in all response rates between the DAC and AZA groups. This finding is consistent with Zhang et al. (2021) and Liu et al.’s studies and a retrospective study of AZA versus DAC in patients with refractory anemia with excess blast (Salim et al., 2016). These differences can be interpreted as follows: a) heterogeneity and publication bias could not be obtained because of the small number of trials investigating each agent; b) our study mainly focused on higher-risk MDS and AML patients, while the previous study included all risk-stratified MDS patients, and the influence of different risk-stratified subgroups cannot be ruled out.

As for the comparisons of high grades AEs for AZA and DAC, previous retrospective studies indicated that patients who received AZA experienced less frequent episodes of grade 3/4 cytopenia and infectious episodes than DAC (Lee et al., 2013a; Lee et al., 2013b). Lee et al. reported more grade 3/4 cytopenia (87 vs. 67%, respectively) and infectious episodes in the DAC group (15.7 cytopenia episodes per 100 cycles vs. 11.8 infectious episodes per 100 cycles) (Lee et al., 2013b). Likewise, Je-Hwan Lee et al. found that high-grade neutropenia occurred more frequently in the DAC group than the AZA group (79.6 vs. 72.2%) (Lee et al., 2013a). Similarly, in our study, DAC demonstrated a higher risk of grade 3/4 anemia, leukopenia, and febrile neutropenia compared with AZA. In our study, we find that, compared with CCR, HMAs demonstrated higher grade 3/4 cytopenia and infectious episodes. This finding is consistent with Gao et al. (2018)’s study.

However, no randomized trial has been ever conducted directly to compare AZA and DAC in AML patients, and a rare randomized trial has been carried out for higher-risk MDS patients. The overwhelming majority of RCTs included in this study were indirect comparisons, and low certainty of the evidence was found when comparing AZA and DAC. Therefore, more head-to-head clinical trials are still required. Additionally, given the limited number of included trials, heterogeneity, network consistency, and publication bias could not be adequately assessed. In studies of MDS patients, we mainly included studies with HR-MDS of more than 60%. Data of some lower-risk patients were also included. This may lead to bias in the results. Optimally, a risk stratification model could be developed to analyze the effects of HMAs in different risk groups. Subgroup analysis could not be assessed due to the paucity of data. This analysis was not robust to sensitivity analyses based on meta-analysis model choice.

## Conclusion

Compared to CCR, AZA and DAC can promote outcomes in patients with AML and HR-MDS. In patients with MDS, DAC demonstrated a higher CR rate than AZA. There were no statistically significant differences between DAC and AZA in other outcomes and in patients with AML. However, AZA experienced lower frequent grade 3/4 leukopenia than patients receiving DAC treatment. For patients with AML or HR-MDS who are unfit for IC or HSCT, both AZA and DAC are available to use. Concerned about the lower hematological toxicity, AZA may be a better choice for elderly patients. However, the available indirect evidence comparing the two agents warrants very low certainty and cannot reliably confirm the superiority of either agent. More head-to-head prospective randomized clinical trials are needed. In the meantime, the choice of either agent should be driven by patients’ preferences, drug availability, and costs.

## Data Availability

The raw data supporting the conclusions of this article will be made available by the authors, without undue reservation.
