# Erratum: Comparison between decitabine and azacitidine for patients with acute myeloid leukemia and higher-risk myelodysplastic syndrome: a systematic review and network meta-analysis

**DOI:** 10.3389/fphar.2023.1213053

**Published:** 2023-05-05

**Authors:** Frontiers Production Office

**Affiliations:** Frontiers Media SA, Lausanne, Switzerland

**Keywords:** decitabine, azacitidine, acute myeloid leukemia, higher-risk myelodysplastic syndrome, network meta-analysis

Due to a production error, there was a mistake in the legends for Figures 6–8 as published. The legend of Figure 6 matches the graph of Figure 7, the legend of Figure 7 matches the graph of Figure 8, and the legend of Figure 8 matches the graph of Figure 6. The correct legends appear below. The publisher apologizes for this mistake.

**FIGURE 6:**
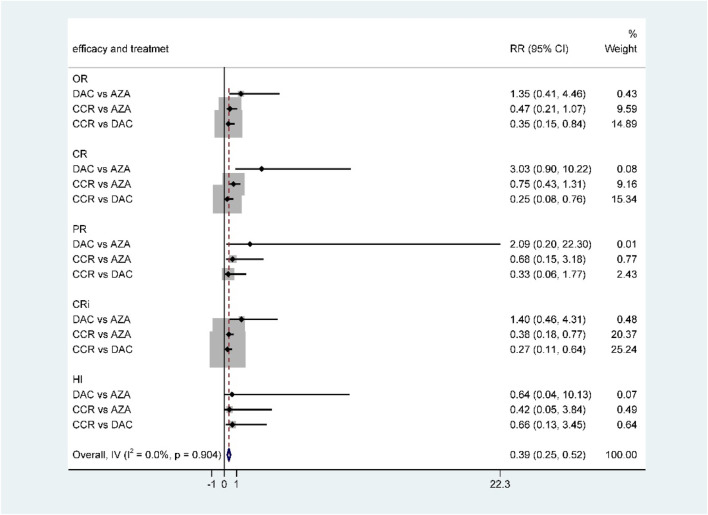
Forest plot of efficacy represents the direct and indirect comparison.

**FIGURE 7:**
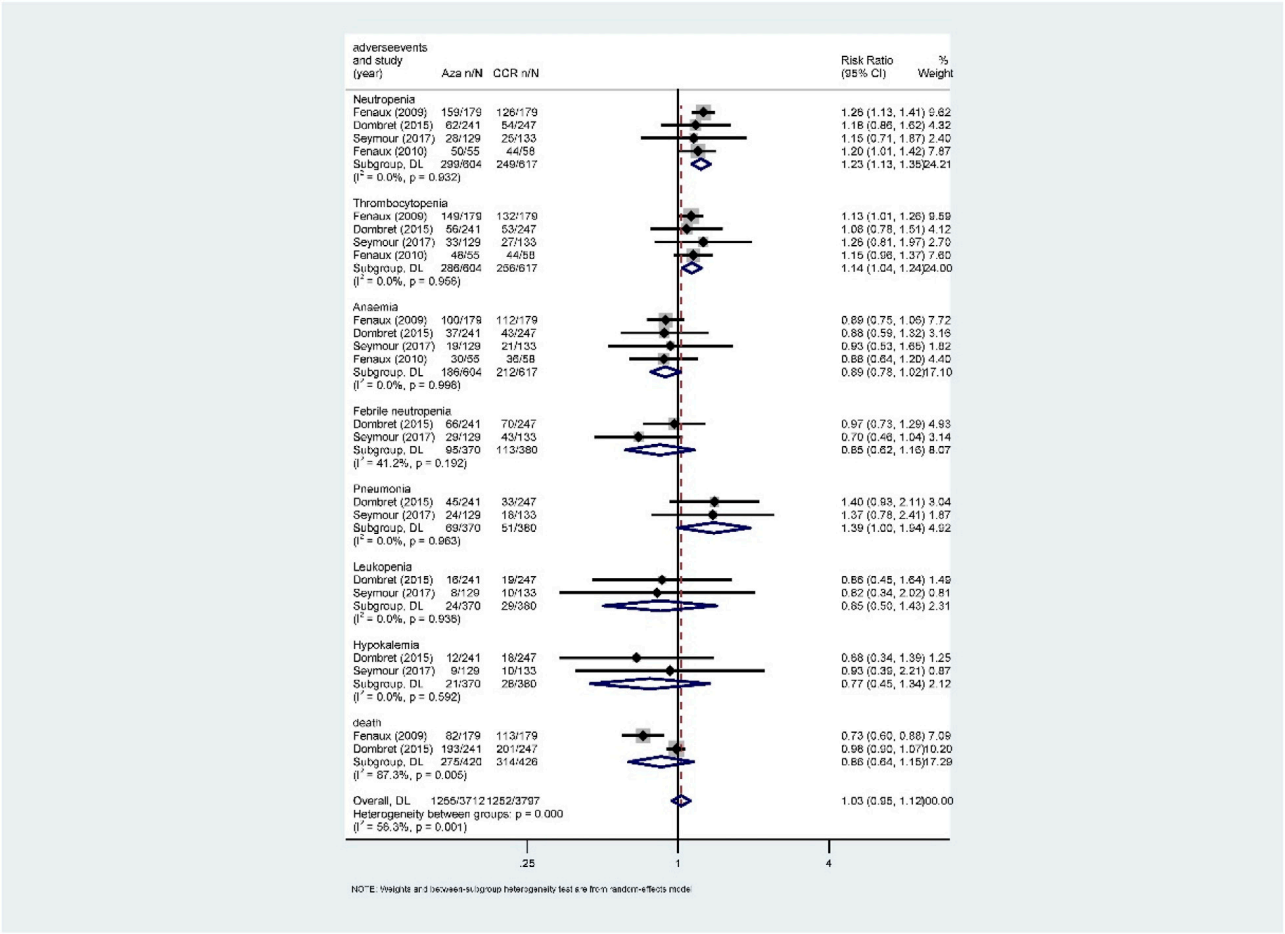
Forest plot of grade 3/4 adverse events of azacitidine vs. conventional care regimens (direct evidence-RR).

**FIGURE 8:**
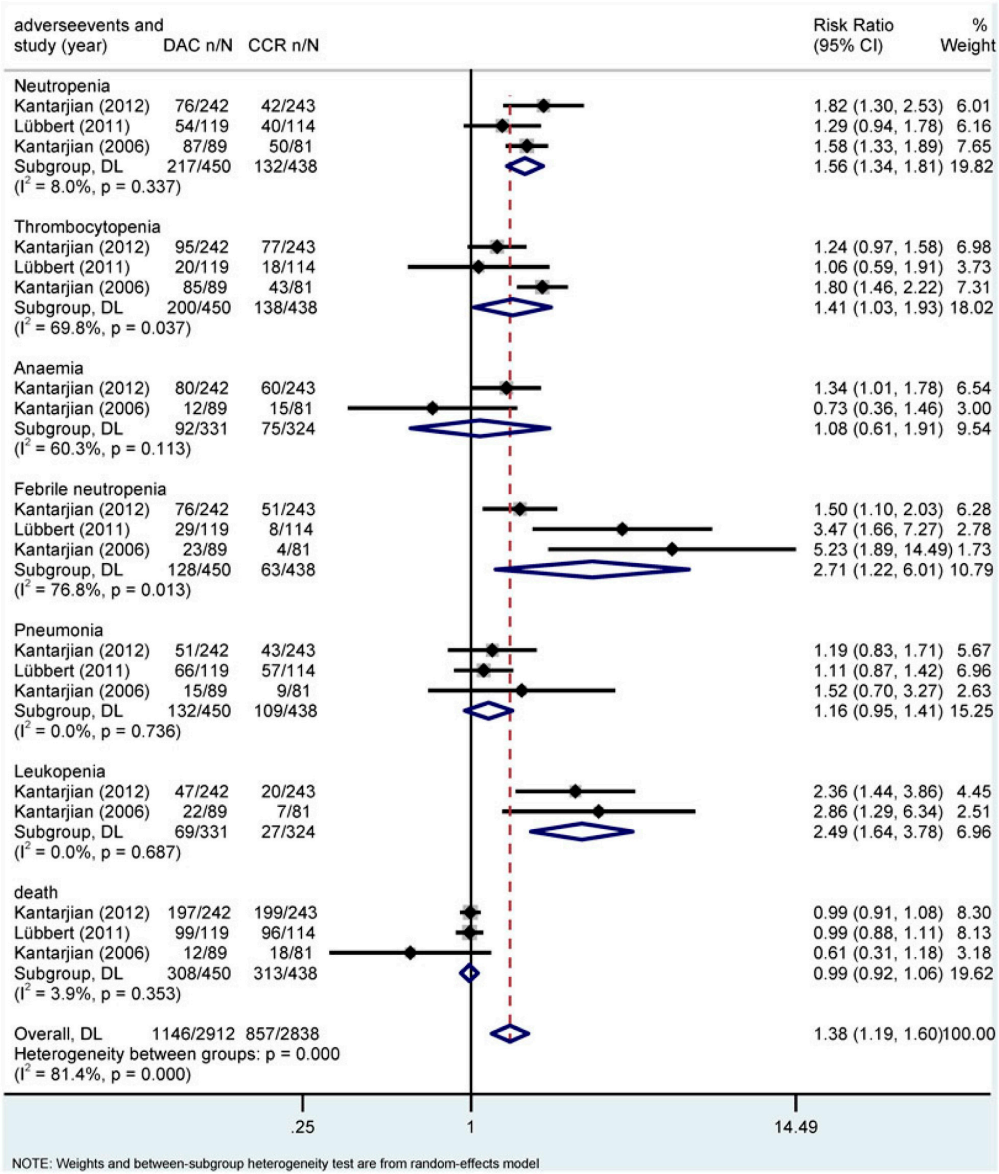
Forest plot of grade 3/4 adverse events of decitabine vs. conventional care regimens (direct evidence-RR).

The original version of this article has been updated.

## Publisher’s note

All claims expressed in this article are solely those of the authors and do not necessarily represent those of their affiliated organizations, or those of the publisher, the editors and the reviewers. Any product that may be evaluated in this article, or claim that may be made by its manufacturer, is not guaranteed or endorsed by the publisher.

