# Supplementary material for: Comparison Between Decitabine and Azacitidine for Patients With Acute Myeloid Leukemia and Higher-Risk Myelodysplastic Syndrome: A Systematic Review and Network Meta-Analysis
Source: Front Pharmacol. 2021 Aug 17;12:701690. doi: 10.3389/fphar.2021.701690 (PMC8416074; doi:10.3389/fphar.2021.701690)
Supplement: Supplementary file 1 [file DataSheet1.PDF]

Supplementary Material

1. Supplementary Figures and Tables

1.1 Supplementary Figures

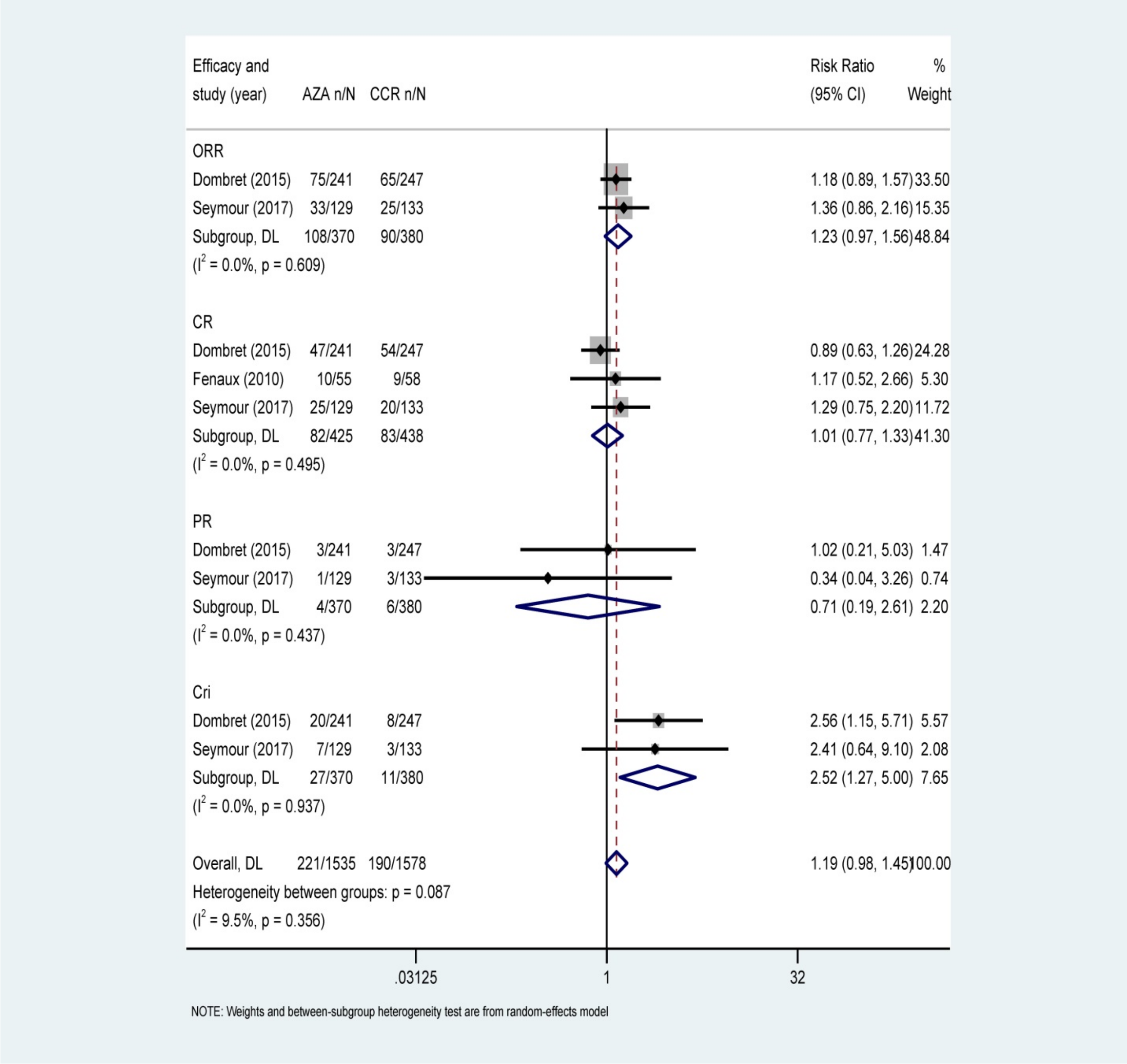

Supplementary Figure 1. Direct comparison of the efficacy of AZA vs. CCR in AML.

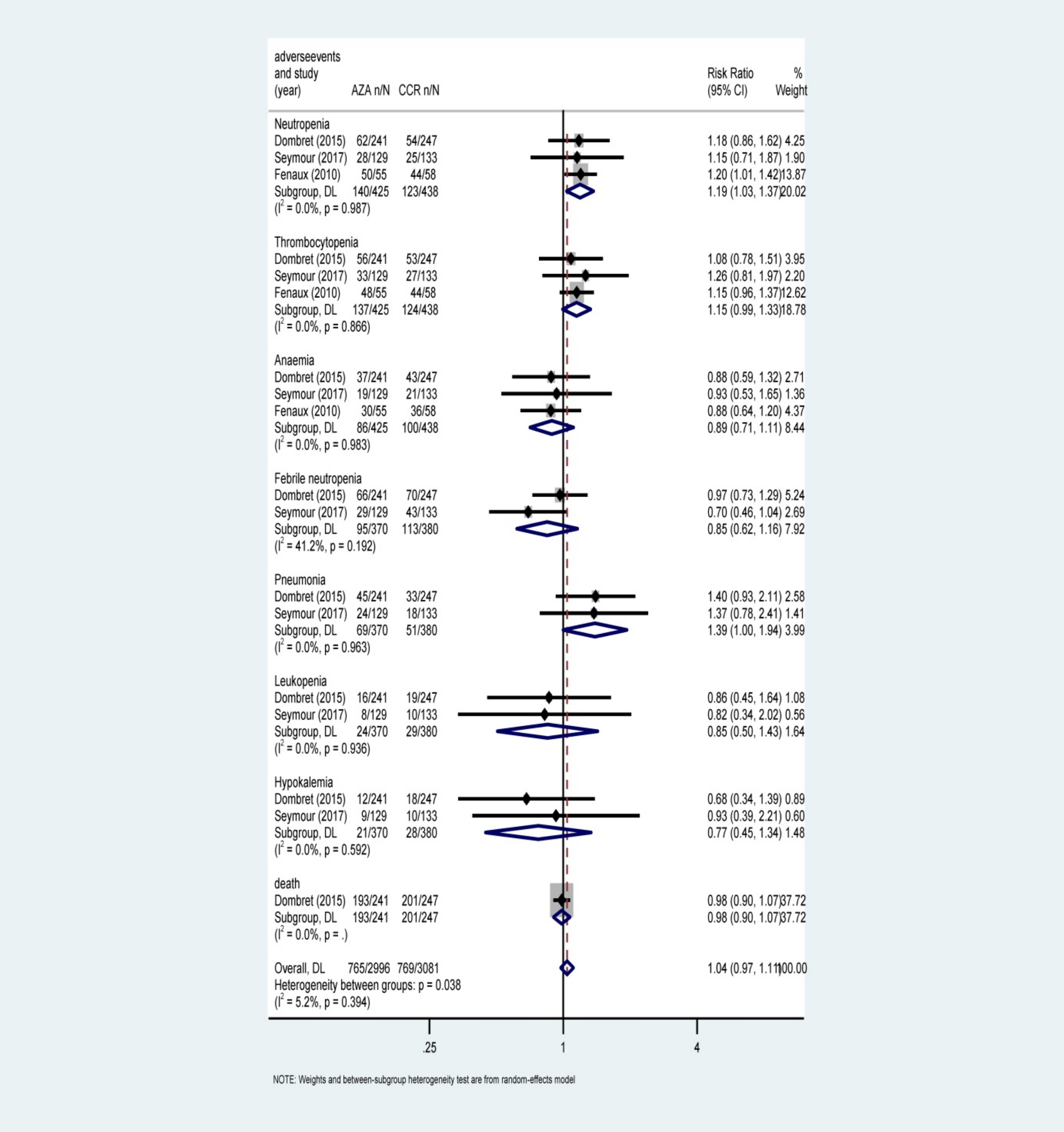

Supplementary Figure 2. Direct comparison of the high grade adverse events of AZA vs. CCR in AML.

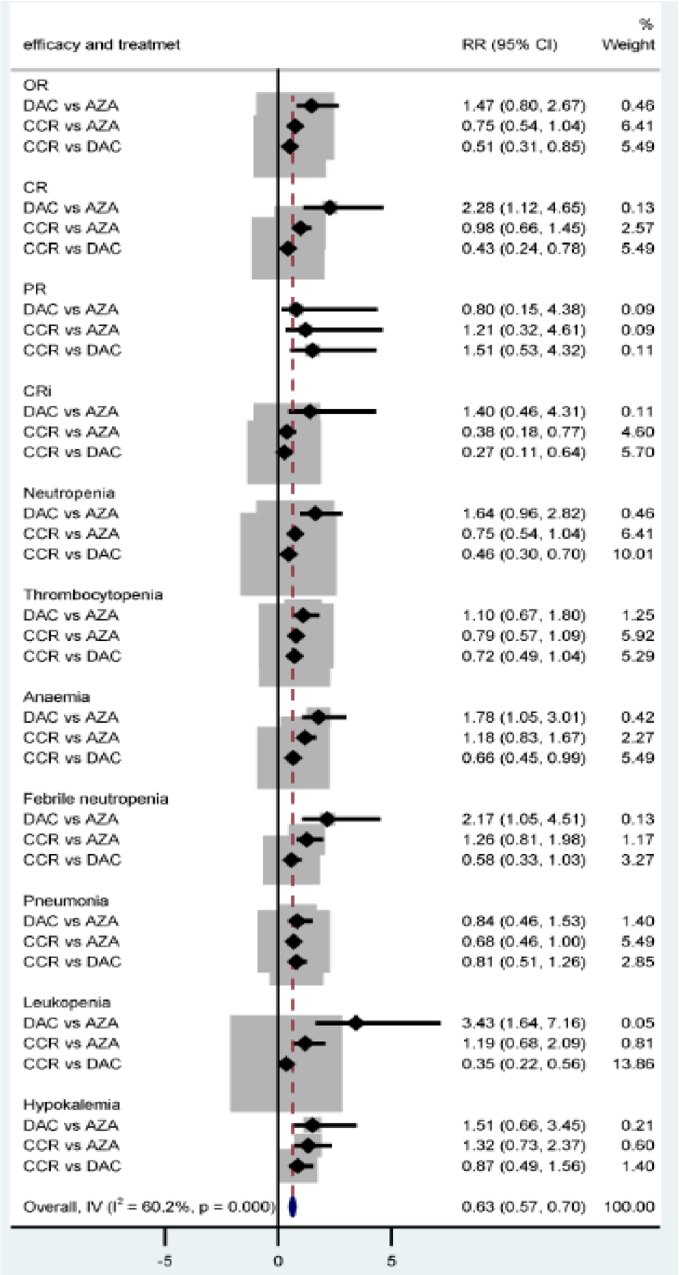

Supplementary Figure 3. Direct and indirect comparison of efficacy and high grade adverse events of AZA vs. CCR in AML.

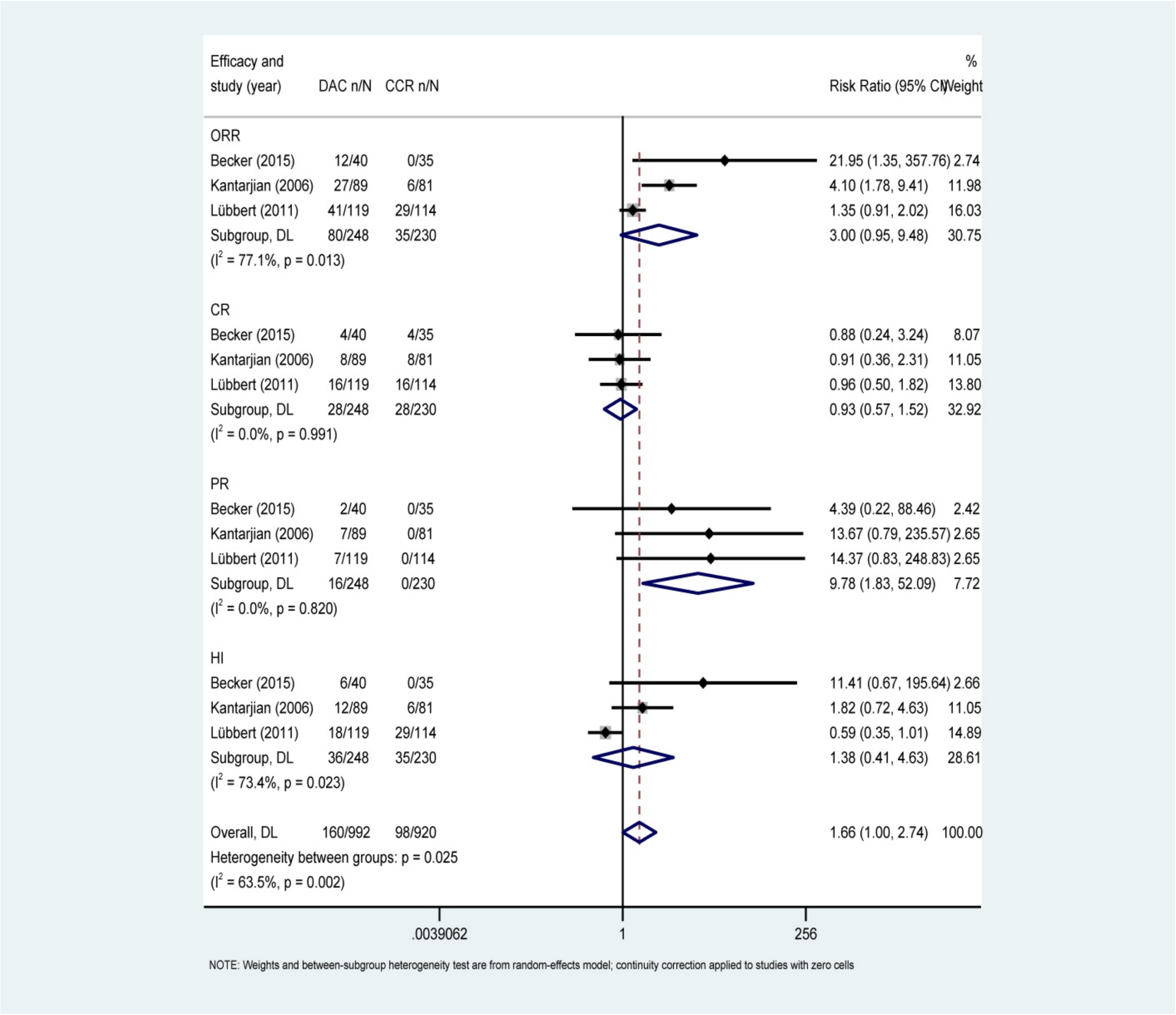

Supplementary Figure 4. Direct comparison of the efficacy of DAC vs. CCR in MDS.

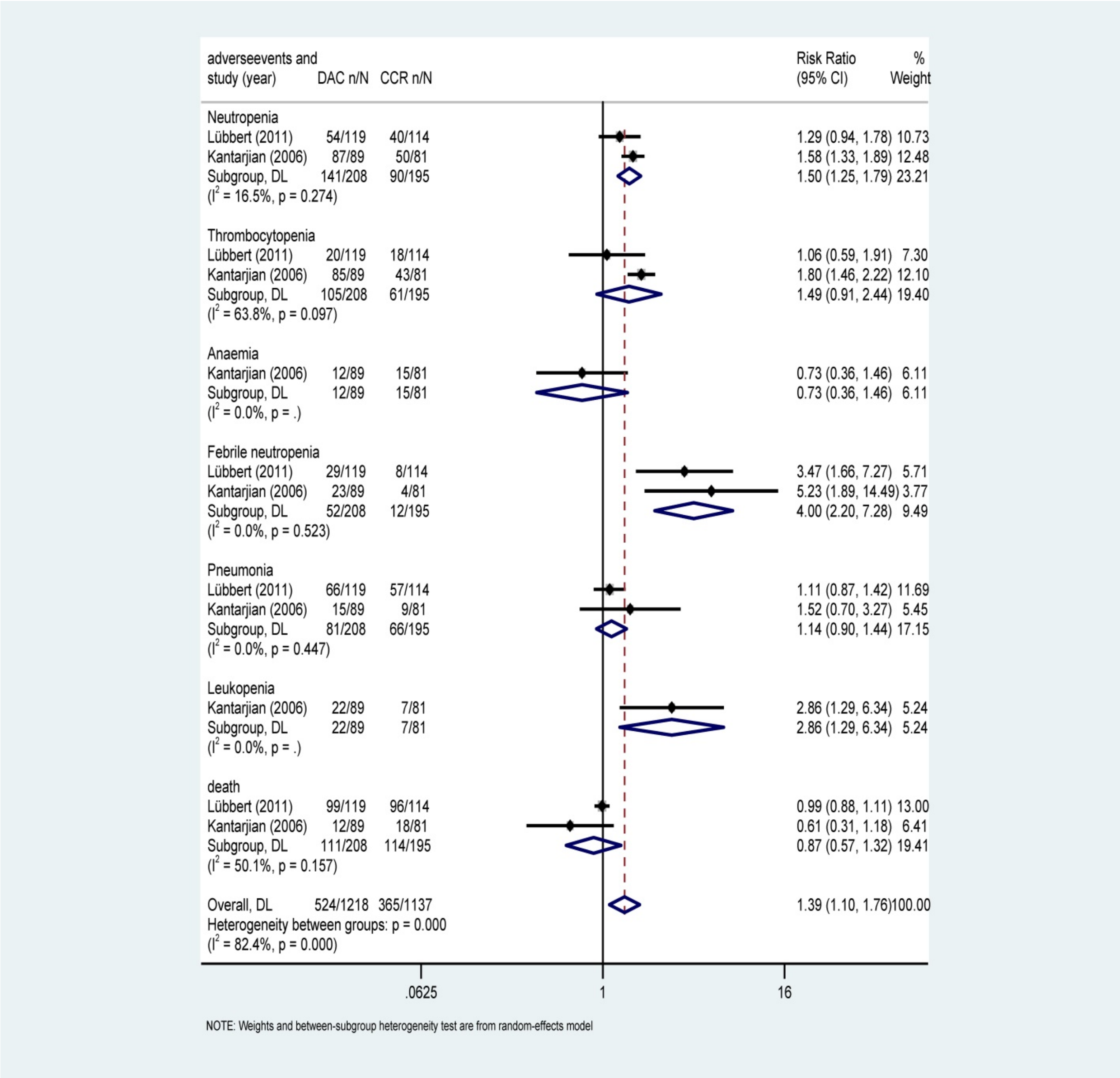

Supplementary Figure 5. Direct comparison of high grade adverse events of DAC vs. CCR in MDS.

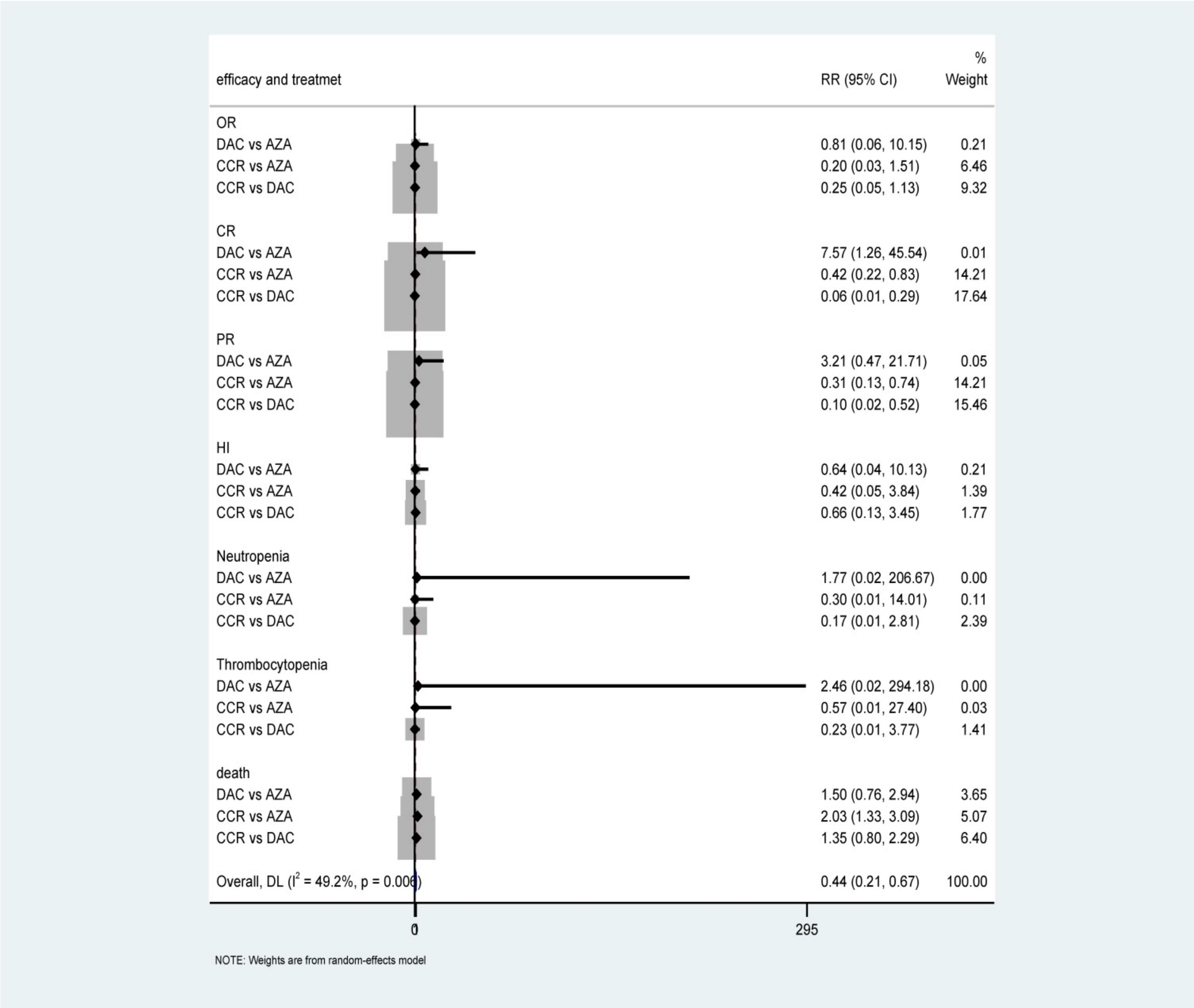

Supplementary Figure 6. Direct and indirect comparison of efficacy and high grade adverse events of DAC vs. CCR in MDS.

1.2 Supplementary tables

Table S1. Search strategies

Pubmed (187)	#1 ("Azacitidine"[Mesh]) OR Azacitidine[Title/Abstract]<br><br>#2 ("Decitabine"[Mesh]) OR Decitabine[Title/Abstract]<br><br>#3 #1 OR #2<br><br>#4 ("Leukemia, Myeloid, Acute"[Mesh]) OR (acute myeloid leukemia [Title/Abstract] OR AML[Title/Abstract])<br><br>#5 (“Myelodysplastic Syndromes” [Mesh]) OR (“myelodysplastic

	syndromes" [Title/Abstract]) OR (MDS [Title/Abstract])<br><br>#6 #4 OR #5<br><br>#7 (random*[Title/Abstract]) OR ("Randomized Controlled Trial"[Publication Type])<br><br>#8 "Randomized Controlled Trial"[Publication Type]<br><br>#9 #7 OR #8<br><br>#10 #3 AND #6 AND #9
Cochrane library (565)	#1 MeSH descriptor: [Azacitidine] explode all trees<br><br>#2 (Azacitidine):ti,ab,kw<br><br>#3 #1 OR #2<br><br>#4 MeSH descriptor: [Decitabine] explode all trees<br><br>#5 ("decitabine"):ti,ab,kw<br><br>#6 #4 OR #5<br><br>#7 #3 OR #6<br><br>#8 MeSH descriptor: [Leukemia, Myeloid, Acute] explode all trees<br><br>#9 ("acute myeloid leukemia"):ti,ab,kw<br><br>#10 #8 OR #9<br><br>#11 MeSH descriptor: [Myelodysplastic Syndromes] explode all trees<br><br>#12 ("Myelodysplastic Syndromes "):ti,ab,kw<br><br>#13 #11 OR # 12<br><br>#14 #10 OR #13<br><br>#15 MeSH descriptor: [Randomized Controlled Trial] explode all trees<br><br>#16 ("random*"):ti,ab,kw<br><br>#17 #15 OR # 16<br><br>#18 #7 AND #14 AND #17

Embase (868)	#1 'azacitidine'/exp<br><br>#2 'azacitidine':ab,ti<br><br>#3 #1 OR #2<br><br>#4 'decitabine'/exp<br><br>#5 'decitabine':ab,ti<br><br>#6 #4 OR #5<br><br>#7 #3 OR #6<br><br>#8 'acute myeloid leukemia'/exp<br><br>#9 'acute myeloid leukemia':ab,ti OR 'AML':ab,ti<br><br>#10 #8 OR #9<br><br>#11 ‘myelodysplastic syndromes’/exp<br><br>#12 ‘myelodysplastic syndromes’:ab,ti OR 'MDS':ab,ti<br><br>#13 #11 OR #12<br><br>#14 #10 OR #13<br><br>#15 'random*':ab,ti<br><br>#16 'randomized controlled trial'/exp<br><br>#17 #15 OR #16<br><br>#18 #7 AND #14 AND #17
Web of science (186)	#1 TS=(Azacytidin*) OR TS=(Azacitidin*) OR TS=(ladakamycin) OR TS=(mylosar) OR TS=(u 18496) OR TS=(Vidaza) OR TS=(NSC-102816) OR TS=(NSC 102816) OR TS=(NSC102816)<br><br>#2 TS=(Deoxycytidine) OR TS=(5-AzadC) OR TS=(AzadC Compound) OR TS=(5AzadC) OR TS=(2'-Deoxy-5-azacytidine) OR TS=(2' Deoxy 5 azacytidine) OR TS=(5-Azadeoxycytidine) OR TS=(5 Azadeoxycytidine) OR TS=(Dacogen) OR TS=(5-Deoxyazacytidine) OR TS=(5 Deoxyazacytidine) OR TS=(NSC 127716) OR TS=(NSC-127716) OR TS=(NSC127716) OR TS=(Decitabine Mesylate)

	#3 #1 OR #2<br><br>#4 TS=(acute granulocytic leukaemia) OR TS=(acute granulocytic leukemia) OR TS=(acute myeloblastic leukaemia) OR TS=(acute myeloblastic leukemia) OR TS=(acute myelocytic leukaemia) OR TS=(acute myelocytic leukemia) OR TS=(acute myelogenous leukaemia) OR TS=(acute myelogenous leukemia) OR TS=(acute myeloid leukaemia) OR TS=(acute nonlymphoblastic leukaemia) OR TS=(acute nonlymphoblastic leukemia) OR TS=(acute nonlymphocytic leukaemia) OR TS=(acute nonlymphocytic leukemia)<br><br>#5 TS=(myelodysplastic syndrome) OR TS=(Syndrome, Myelodysplastic) OR TS=(Syndromes, Myelodysplastic) OR TS=(Dysmyelopoietic Syndromes) OR TS=(Dysmyelopoietic Syndrome) OR TS=(Syndrome, Dysmyelopoietic) OR TS=(Syndromes, Dysmyelopoietic) OR TS=(Hematopoetic Myelodysplasia) OR TS=(Hematopoetic Myelodysplasias) OR TS=(Myelodysplasia, Hematopoetic)<br><br>#6 #4 OR #5<br><br>#7 ALL=(randomised controlled study) OR ALL=(randomised controlled trial) OR ALL=(randomized controlled study) OR ALL=(random*) OR ALL=(RCT)<br><br>#8 #3 AND #6 AND #7

Table S2. Estimation of NMA inconsistency: summary of results of Aza vs Dec

Outcome and data	Number of studies	P-value of local inconsistency (node-splitting)
Overall response	7	7
Complete remission	8	8
Partial remission	7	7
Complete remission with incomplete blood count recovery	3	0.999
Hematology improvement	4	0.994
Neutropenia	7	0.998

Thrombocytopenia	7	1.000
Anaemia	6	0.995
Febrile neutropenia	5	0.984
Pneumonia	5	0.995
Leukopenia	4	0.989
Hypokalemia	3	0.997
Death	5	0.991

Table S3. Estimation of NMA consistency: summary of results of Aza vs Dec

Outcome and data	Number of studies	Coef.(LogRR)	Std. Err.	P-value of the design by treatment test	[95% Conf. Interval]
Overall response rate	7	0.299	0.611	0.625	-0.89 – 1.49
Complete remission	8	1.109	0.620	0.074	-0.11 – 2.32
Partial remission	7	0.739	1.207	0.54	-1.63 – 3.10
Complete remission with incomplete blood count recovery	3	0.339	0.572	0.553	-0.78 – 1.46
Hematology improvement	4	-0.186	1.194	0.876	-2.53 – 2.15
Neutropenia	7	0.234	0.083	0.005	0.07 – 0.39

Thrombocytopenia	7	0.549	0.701	0.434	-0.82 – 1.92
Anaemia	6	0.474	0.228	0.037	0.03 – 0.92
Febrile neutropenia	5	1.393	0.536	0.009	0.34 – 2.44
Pneumonia	5	-0.139	0.258	0.589	-0.65 – 0.36
Leukopenia	4	1.074	0.341	0.002	0.41 – 1.74
Hypokalemia	3	0.380	0.386	0.324	-0.38 – 1.14
Death	5	0.099	0.143	0.489	-0.18 – 0.38

Table 1. Characteristics of publications

Study, year	Type	Intervention (dose, schedule)	Patients enrolled	Female	Age, median	WHO classification	FAB classification	IPSS	Karyotape risk	ECOG	Median cycles	Efficacy	Grade3/4) adverse events	Median OS (months)
Fenaux et al. 2009	Phase III	Aza (75 mg/m2/d*7d per 28-day cycle for at least 6 cycles)	179	47	69 (42–83)	RAEB-1 14, RAEB-2 98, CMML-1 1, CMML-2 10, AML 55, Indterminate 1	RAEB 104，RAEB-T 61，CMML 6, AML 1	IPSS-1 5, IPSS-2 76, High 82	Favorable 83, Intermediate 37, Unfavorable 50, unknown 9	0: 78, 1: 86, ≥2: 13, unknown: 2	9 (4-15)	OR 138, CR 30, PR 21, HI 87	Neutropenia 159, thrombocytopenia 149, anaemia 100, death 82	24.5
		CCR (BSC 105, LDA 49, intensive chemotherapy 25)	179	60	70 (38–88)	RAEB-1 17, RAEB-2 95, CMML-1 0, CMML-2 5, AML 58, Indterminate 4	RAEB 103，RAEB-T 62，CMML 5, AML 1	IPSS-1 13, IPSS-2 70, High 85	Favorable 84, Intermediate 39, Unfavorable 50, unknown 6	0: 80, 1: 86, ≥2: 10, unknown: 3	NA	OR 72, CR 14, PR 7, HI 51	Neutropenia 126, thrombocytopenia 132, anaemia 112, death 113	15
Dombret et al.	Phase III	Aza (75	241	102	75(64-91)	AML	AML	-	-		6 (1-28)	OR 75,	Febrile neutropenia 66, pneumonia	10.4

2015		mg/m2/d*7d per 28-day cycle for at least 6 cycles)										Cri 20,<br><br>CR 47,<br><br>PR 3	45, leukopenia 16, hypokalemia 12,<br><br>neutropenia 62, thrombocytopenia 56, anaemia 37, death 193	
		CCR (BSC, LDAC<br><br>(20 mg bid *10d per 28-day treatment cycle for at least 4 cycles),IC (Ara-c100-200 mg/m2/d *7d, daunorubicin 45-60 mg/m2/d*3d or<br><br>idarubicin 9-12	247	98	75(65-89)	AML	AML	-	-	-	2 (1-3) IC cycles, and 4 (1-25) LDAC cycles, and the median exposure to BSC only was 65 (6-535) days	OR 65,<br><br>Cri 8,<br><br>CR 54,<br><br>PR 3	Febrile neutropenia 70, pneumonia 33, leukopenia 19, hypokalemia 18,<br><br>neutropenia 54, thrombocytopenia 53, anaemia 43, death 201	6.5

		mg/m2/d*3d)												
Kantarjian et al. 2012	Phase III	Dec (20 mg/m(2)/d*5 d per 28-day cycle)	242	105	73(64-89)	AML	AML	-	Favorable NA, Intermediate 152, Unfavorable 87, unknown NA	1: 184, ≥2: 58	NA	OR 49, Cri 24, CR 38, CRp 5, PR 6	Febrile neutropenia 76, pneumonia 51, leukopenia 47, hypokalemia 27, neutropenia 76, thrombocytopenia 95, anaemia 80, dyspnea 16, death 197	7.7
		CCR (BSC or cytarabine 20 mg/m(2)/d as a subcutaneous injection for 10 consecutive days every 4 weeks)	243	92	73(64-91)	AML	AML	-	Favorable NA, Intermediate 154, Unfavorable 87, unknown NA	1: 183, ≥2: 60	NA	OR 28, Cri 7, CR 18, CRp 1, PR 9	Febrile neutropenia 51, pneumonia 43, leukopenia 20, hypokalemia 24, neutropeni	5

													a 42, thrombocytopenia 77,<br><br>anaemia 60,<br><br>dyspnea 14, death 199	
Lübbert et al. 2011	Phase II	Dec (15 mg/m2 q8h *3 d,every 6-week cycles)	119	43	69(60-90)	MDS	RAEB 61,RAEB-T 40,CMML 10, AML 1	IPSS-1 8, IPSS-2 64, High 46, unknown 1	Favorable 38, Intermediate 8, Unfavorable 57, unknown 15	0: 29,<br><br>1: 76,<br><br>≥2: 14,	4	OR 41,<br><br>CR 16,<br><br>PR 7, HI 18	Febrile neutropenia 29, pneumonia 66, neutropenia 54, thrombocytopenia 20,<br><br>anaemia NA,<br><br>death 99	10.1
		BSC	114	41	70(60-86)	MDS	RAEB 64,RAEB-T 35,CMML 4, AML 1	IPSS-1 8, IPSS-2 63, High 42, unknown 1	Favorable 29, Intermediate 17, Unfavorable 51, unknown 17	0: 25,<br><br>1: 72,<br><br>≥2: 17	NA	OR 28,<br><br>Cri 7,<br><br>CR 18,<br><br>CRp 1,	Febrile neutropenia 8, pneumonia 57, neutropenia 40, thrombocyt	8.5

												PR 9	openia 18, anaemia NA, death 96	
Seymour et al.2017	RCT	Aza (75 mg/m2/d*7d per 28-day cycle)	129	48	76 (64-90)	AML	AML	-	Favorable NA, Intermediate 63, Unfavorable 66,	1: 94, ≥2: 35	5(1-27)	OR 33, Cri 7, CR 25, PR 1	Febrile neutropenia 29, pneumonia 24,, leukopenia 8, hypokalemia 9, neutropenia 28, thrombocytopenia 33, anaemia 19, dyspnea 6, sepsis 7, death NA	8.9
		CCR	133	55	75 (65-87)	AML	AML	-	Favorable NA, Intermediate 61, Unfavorable 72,	1: 104, ≥2: 29	2	OR 25, Cri 3, CR 20,	Febrile neutropenia 43, pneumonia 18,, leukopenia	4.9

												PR 2	10, hypokalemia 10,<br><br>neutropenia 25, thrombocytopenia 27,<br><br>anaemia 21,<br><br>dyspnea 4, sepsis 9, death NA	
Fenaux et al. 2010	Phase III	Aza (75 mg/m2/d*7d per 28-day cycle for at least 6 cycles)	55	18	70(52-80）	AML	AML	-	Favorable 19, Intermediate 38, Unfavorable 14, unknown 3	0: 16,<br><br>1: 35,<br><br>≥2: 4, unknown 0	NA	CR 10	Neutropenia 50, thrombocytopenia 48,<br><br>anaemia 30,<br><br>death NA	24.5
		CCR	58	17	70(50-83)	AML	AML	-	Favorable 33, Intermediate 43, Unfavorable 13, unknown 2	0: 22,<br><br>1: 34,<br><br>≥2: 0, unknown 2	NA	CR 9	Neutropenia 44, thrombocytopenia 44,<br><br>anaemia 36,	16

													death NA	
Kantarjian et al. 2006	Phase III	Dec(15mg/m2 q8h*3d,every 6 weeks)	89	30	70(65-76)	NA	RA 12, RARS 7, RAEB 47, RAEB-T 17, CMML 6	IPSS-1 28, IPSS-2 38, High 23	NA	0: 21,<br>1: 61,<br>≥2: 4, unknown 0	3(0-9)	OR 27,<br>CR 8,<br>PR 7,<br>HI 12	Febrile neutropenia 23, pneumonia 15, leukopenia 22,<br>neutropenia 87, thrombocytopenia 85,<br>anaemia 12,<br>death 12	14
		BSC	81	24	70(62-74)	NA	RA 12, RARS 4, RAEB 43, RAEB-T 14, CMML 8	IPSS-1 24, IPSS-2 36, High 21	NA	0: 28,<br>1: 48,<br>≥2: 4, unknown 1	NA	OR 6,<br>HI 6	Febrile neutropenia 4, pneumonia 9, leukopenia 7,<br>neutropenia 50, thrombocytopenia 43,<br>anaemia	14.9

													15,<br>death 18	
Becker et al.2015	Phase III	Dec (15 mg/m2 q8h *3 d,every 6-week cycles)	40	11	69.5(61-90)	MDS	RAEB-t	IPSS-1 2, IPSS-2 12, High 26	Favorable 16, Intermediate 4, Unfavorable 14, unknown 6	0: 8,<br>1: 29,<br>≥2: 3	NA	OR 12,<br>CR 4,<br>PR 2,<br>HI 6	NA	8
		BSC	35	11	69(61-80)	MDS	RAEB-t	IPSS-1 0, IPSS-2 13, High 22	Favorable 5, Intermediate 5, Unfavorable 17, unknown 8	0: 10,<br>1: 19,<br>≥2: 6	NA	OR 0,<br>CR 0,<br>PR 0,<br>HI 0	NA	6
